# Latent brain–cognition dimension indicative of neuroinflammatory basis of Long COVID

**DOI:** 10.1093/braincomms/fcag344

**Published:** 2026-09-19

**Authors:** Pavel Filip, Eliška Bartečková, Jana Hořínková, Patrik Bartys, David Ulčák, Martin Radvan, Gabriela Matejová, Irena Rektorova, Shalom Michaeli, Silvia Mangia, Lubomír Vojtíšek

**Affiliations:** Department of Neurology, Charles University, First Faculty of Medicine and General University Hospital, Prague 128 08, Czech Republic; Department of Cybernetics, Czech Technical University in Prague, Prague 160 00, Czech Republic; Center for Magnetic Resonance Research (CMRR), University of Minnesota, Minneapolis, MN 55455, USA; Department of Psychiatry, Faculty of Medicine, Masaryk University and University Hospital Brno, Brno 625 00, Czech Republic; Department of Psychiatry, Faculty of Medicine, Masaryk University and University Hospital Brno, Brno 625 00, Czech Republic; Department of Psychiatry, Faculty of Medicine, Masaryk University and University Hospital Brno, Brno 625 00, Czech Republic; Central European Institute of Technology, Masaryk University, Brno 625 00, Czech Republic; Department of Internal Medicine and Cardiology, University Hospital Brno and Faculty of Medicine of Masaryk University, Brno 625 00, Czech Republic; Department of Internal Medicine and Cardiology, University Hospital Brno and Faculty of Medicine of Masaryk University, Brno 625 00, Czech Republic; Central European Institute of Technology, Masaryk University, Brno 625 00, Czech Republic; Center for Magnetic Resonance Research (CMRR), University of Minnesota, Minneapolis, MN 55455, USA; Center for Magnetic Resonance Research (CMRR), University of Minnesota, Minneapolis, MN 55455, USA; Central European Institute of Technology, Masaryk University, Brno 625 00, Czech Republic

**Keywords:** MRI, Long COVID, relaxometry, diffusion-weighted imaging

## Abstract

Long COVID is characterized by persistent multisystemic symptoms with poorly defined pathophysiological substrate. The presented study investigated whether Long COVID is associated with alterations in brain microstructure and cognition by jointly modelling multimodal MRI and neuropsychological data within a shared framework. One hundred nine participants (77 Long COVID, 32 controls) without the history of severe acute COVID infection underwent quantitative MRI (T_1_, T_2_, T_1_ρ, T_2_ρ relaxometry, neurite orientation dispersion and density indices derived from diffusion-weighted imaging) and neuropsychological testing. The resulting brain–cognition axis provided a robust group separation across dimensionalities (permutation *P* ≈ 0.02), with 72.6% of variance attributable to cognition and 27.4% to MRI features. The predominant imaging features included higher isotropic free-water and reduced neurite density, with minimal relaxometry influence. Projection of discriminant weights back to anatomy revealed involvement over cortico-subcortical dorsal attention, salience and limbic circuits, consistent with patients’ cognitive complaints. Furthermore, brain–cognition axis score correlated not only with the global Long COVID symptom burden but also with its respiratory, systemic, and neurological domains without clear predominance for any symptom group (partial *R*^2^ ≈ 0.10–0.18, *q* < 0.01). Hence, although the cognitive profile provided the dominant discriminative anchor in group separation, neuroimaging offered complementary information and pathophysiological background for the clinically apparent cognitive phenotype. The prominence of neuroinflammation-sensitive MRI protocols highlights probable immune and vascular contributions rather than overt neurodegeneration as the basis of Long COVID in individuals without a severe acute infection course.

## Teaser

Filip *et al*. describe long COVID as a complex entity involving cognitive impairment linked with altered cerebral microstructure. The prominence of neuroinflammation-sensitive MRI protocols in the jointly modelled latent axis points to diffuse immune and vascular contributions rather than neurodegenerative substrate.

## Graphical Abstract

**Graphical Abstract:**
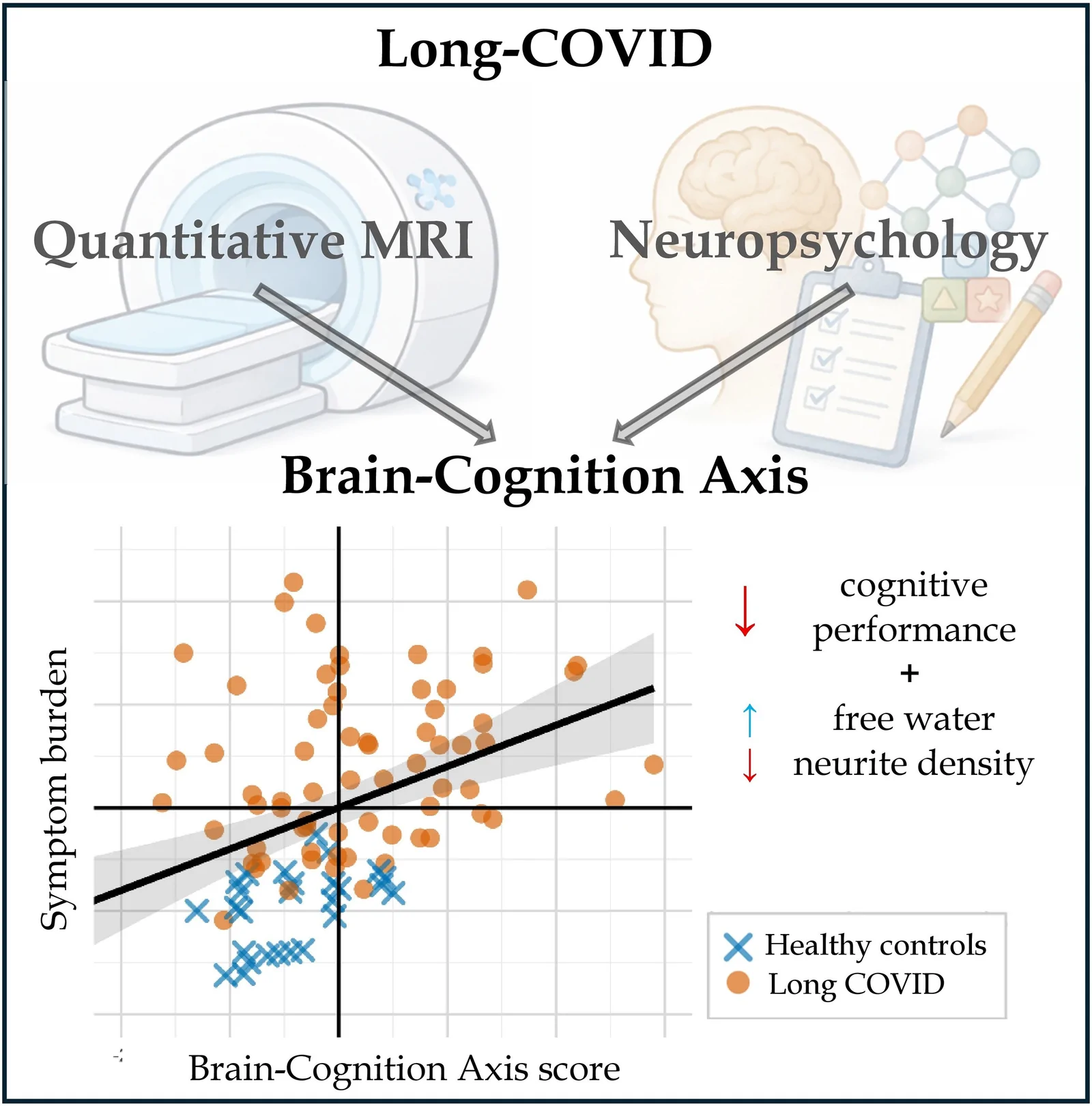

## Introduction

The global pandemic of severe acute respiratory syndrome coronavirus 2 (SARS-CoV-2) was overcome only at great cost but has left the public health facing a new challenge—a wide spectrum of protracted symptoms persisting beyond the acute phase of SARS-CoV-2 infection referred to as Long COVID, post-COVID-19 condition or post-COVID syndrome.^1^ Several per cent of previously infected individuals are thought to be affected, although prevalence varies substantially depending on case definition, viral variant and follow-up duration.^2^ The clinical phenotype is heterogeneous, ranging from fatigue, sleep disturbances, diffuse pain and dyspnoea to cognitive complaints, anxiety and depression.^3^ Multiple, likely overlapping mechanisms have been hypothesized, including immune dysregulation,^4^ viral antigen persistence,^5^ latent herpesvirus reactivation,^6^ endothelial and microvascular dysfunction^7^ and disruption of the gut–brain axis.^8^

Neuroimaging studies have expanded substantially since the earliest case reports. The acute phase of COVID-19 has been associated with hypoperfusion, white-matter hyperintensities, microhaemorrhages and ischaemic lesions, particularly in patients with severe illness.^9,10^ Even individuals without persisting subjective complaints after recovery from acute infection may not be entirely spared, as longitudinal analyses revealed orbitofrontal and parahippocampal cortical atrophy accompanied by cognitive decline.^11^

In Long COVID, the emerging picture is more heterogeneous and subtle. Structural,^12,13^ hypometabolic^14^ and hypoperfusion alterations^15^ within limbic, olfactory and association cortices have been reported, implicating network-wide neurovascular dysregulation. White-matter changes compatible with neuroinflammation, including oedema, demyelination and axonal injury, have also been described.^16,17^ Yet, other cohorts, including several studies of predominantly non-hospitalized patients, have reported minimal or absent changes to brain microstructure despite pronounced subjective complaints.^18-20^ Altogether, Long COVID seems to emerge as a disorder not defined by overt focal damage, but rather diffuse and more subtle alterations to the nervous system.

Indeed, analyses confined to an isolated imaging modality or restricted pools of cognitive or behavioural determinants will inherently underestimate the complexity of the underlying processes, particularly with the subtle and distributed nature of alterations. Multimodal quantitative MRI overcomes this limitation by providing complementary perspectives: free-precession longitudinal (T_1_) and transversal (T_2_) relaxation times in combination with adiabatic rotating-frame relaxation metrics (T_1_ρ and T_2_ρ) offer indirect estimates of myelin density,^21^ iron load,^22^ and integrity of biological processes such as enzymatic activity and water–protein interactions linked to cellular metabolism.^23,24^ Neurite Orientation Dispersion and Density Imaging (NODDI) as a biologically validated marker of dendritic complexity, neurite integrity and oedema^25^ extends the picture into an interlocking overview of microstructural organization and neuroinflammatory alterations, beyond the possibilities of single-modality studies. These sources of information must be considered jointly with cognitive dimensions to avoid fragmenting the evidence and overlooking clinically meaningful, high-level patterns.

In the presented study, we introduced the concept of the brain–cognition axis as a joint latent dimension designed to capture the shared variance between cerebral microstructure and objective and subjective cognitive performance measures. Within this framework, our objectives were (i) to establish whether Long COVID is associated with a shift along the domain integrating brain microstructure and cognitive performance; (ii) to localize eventual neurobiological substrates of this alteration and identify circuits most affected in Long COVID; and (iii) to evaluate whether the variation along the brain–cognition axis reflects the severity of persisting Long COVID symptoms or a specific Long COVID phenotype. We hypothesized that Long COVID involves mild but distributed microstructural alterations across multiple circuits relevant for cognition, sensorimotor function and autonomic regulation, accompanied by reduced memory and executive performance.

## Materials and methods

### Participants

A total of 162 individuals with a documented history of COVID-19 infection were enrolled at the Central European Institute of Technology in cooperation with the University Hospital Brno, Czech Republic, based on a public campaign between 2021 and 2023. The cohort included participants with persisting post-infectious complaints as well as individuals without long-term sequelae. Participants with persistent complaints were examined by a specialist in internal medicine to exclude other somatic conditions that could account for their symptoms. Basic demographic information, clinical details related to COVID-19 (symptoms during acute infection, time elapsed since infection, history of COVID-19-related hospitalization and mechanical ventilation) and responses to the Newcastle Post-COVID Syndrome Follow-Up Screening Questionnaire (14 items) were collected contemporary with MRI acquisition, therefore representing the clinical status at that time. The exclusion criteria comprised a history of severe COVID-19 infection course requiring artificial pulmonary ventilation and/or intensive-care unit monitoring (hospitalization at a standard ward level was permitted), standard contraindications to MRI, comorbid neurological disorders and pregnancy.

Every participant provided a written informed consent in accordance with the Declaration of Helsinki. The study protocol was approved by the ethics committee of the Faculty of Medicine, Masaryk University.

### MRI and neuropsychological data acquisition

MRI acquisition was performed using a 3 Tesla Siemens MAGNETOM Prisma with a 64-channel head-neck coil at the Central European Institute of Technology in Brno, Czech Republic. Whole-brain coverage including the cerebellum and brainstem was obtained.

- T_1_-weighted/T_1_ map (MP2RAGE): isotropic 1.0 mm^3^; repetition time (TR)/echo time (TE)/inversion times TI_1_/TI_2_ = 5000/2.98/700/2500 ms; flip angle (FA) 4°/5°; GRAPPA 3.^26^
- T_2_-weighted (SPACE): isotropic 1.0 mm^3^; TR/TE = 3200/412 ms; GRAPPA 2.
- rs-fMRI (MB-EPI—bold): isotropic 2.0 mm^3^; TR/TE = 820/35.2 ms; FA 45°; MB 4; 500 volumes; two runs with reversed phase-encoding (AP, PA). Spin-echo fieldmaps (AP/PA) were acquired for distortion correction.
- DWI: (MB-EPI DIFF) isotropic 1.5 mm^3^; TR/TE = 3222/89.2 ms; MB 4; two shells *b* = 1500/3000 s/mm^2^, 93 directions, 7 interleaved *b*_0_ volumes; acquired twice with reversed phase-encoding (AP, PA).^27^
- T_2_ map: voxel size 1.0 × 1.0 × 3.5 mm^3^; TR/TE = 7020/10–170 ms (17 echoes, ΔTE 10 ms); GRAPPA 2.
- Adiabatic T_1_ρ/T_2_ρ: (GRE-prep) voxel size 1.6 × 1.6 × 3.5 mm^3^; TR/TE = 2000/2.82 ms; GRAPPA 3; adiabatic full-passage (AFP) pulses (*R* = 10, duration 6 ms; 0–16 pulses phase-cycled by MLEV-4; bandwidth 1.3 kHz; ω_1_max/(2π) = 800 Hz). For T_2_ρ, this train was bracketed by adiabatic half-passage (AHP) pulses.^28,29^

Neuropsychological testing was performed in person by trained examiners in the native language of the participants (Czech). The battery included the Rey–Osterrieth Complex Figure Test (copy, immediate and delayed recall), the Auditory Verbal Learning Test (AVLT), the Brief Visuospatial Memory Test–Revised (BVMT), verbal fluency, object classification, the Trail Making Test parts A and B, and the Mazes test. Subjective cognitive difficulties were additionally assessed using the Cognitive Failures Questionnaire (CFQ).

### Clinical and cognitive data processing

For the purposes of analysis, participants were stratified into two groups. The long COVID group comprised individuals who either reported ongoing subjective problems after COVID-19 or specified at least two additional items in the Newcastle questionnaire (Q2–14), with symptoms persisting for at least 2 months after the acute infection. The control group (HC) included participants without any complaints (<2 items from Newcastle questionnaire 2–14).

Raw Newcastle score was calculated as the sum of endorsed items. Separately, a pre-specified three-factor confirmatory factor model was fitted with latent domains of *respiratory complaints* (Q2 cough, Q4 breathlessness, Q5 chest pain), *systemic symptoms* (Q3 fatigue, Q6 fever, Q7 myalgia, Q10 sleep disturbance, Q13 mood) and *neurological/sensory–autonomic features* (Q8 headache, Q9 concentration, Q11 taste, Q12a smell, Q12b dizziness), allowing one residual covariance (Q8∼Q9). Items were treated as ordered categorical variables, estimation used the robust weighted least squares-mean and variance adjusted estimator with theta parameterization, and latent variances were standardized.

Raw (non-norm-referenced) neuropsychological measures were utilized since demographic effects were jointly modelled across all modalities. However, to evaluate the influence of this choice on study conclusions, a robustness analysis was performed using norm-referenced neuropsychological scores. Data were harmonized to code better performance by higher values (inverse transformation of Trail Making Test A and B completion times) and *z-*standardized across the full sample. A Bayesian confirmatory factor model was specified with two latent cognitive constructs: *Memory* (Rey figure recall trials, AVLT total, overall and delayed recall, BVMT total, overall and delayed recall) and *Executive function* (Rey copy, verbal fluency, oral control, Trail Making A and B, Mazes). Residual covariances were specified among subtests from the same instrument families (AVLT, BVMT, Rey test). Model estimation was performed under a Bayesian framework using Hamiltonian Monte Carlo with four independent chains, each run with 2000 burn-in and 4000 retained sampling iterations (16 000 posterior draws in total). Posterior draws were collated across parameters and convergence assessed using the potential scale reduction factor (considered adequate if all $\hat{R}$ ≤ 1.01) and effective sample sizes (adequate if ≥ 1000). The overall fit was examined via posterior predictive checks, including the posterior predictive *P*-value. Lastly, Memory and Executive factor scores were extracted as posterior means and re-oriented to ensure positive association with higher task performance (anchored to AVLT total score and TMT-B, respectively).

### Image preprocessing

The custom processing pipeline was built around the Human Connectome Project (HCP) minimal preprocessing pipeline, preceded by denoising of the T_1_-weighted scan derived from MP2RAGE,^30^ DWI data denoising based on random matrix theory and Marchenko–Pastur distribution^31^ with the optimized patch-based singular value shrinkage method^32^ and Gibbs ringing artefacts removal utilizing the local subvoxel shifts method.^33^ The HCP minimal preprocessing pipeline was implemented including the optional correction of gradient non-linearity and susceptibility-induced B_0_-field distortions and the HCP rs-fMRI pipeline.^34^ rs-fMRI output was utilized only for multimodal areal feature-based surface registration algorithm (MSMAll), no other rs-fMRI metrics were derived. Voxel-wise fibre orientation distributions were estimated from DWI data with GPU-accelerated Bayesian sampling, implementing axially symmetric Gaussian (‘zeppelin’) single-fibre response (up to three fibre populations per voxel), a burn-in of 3000 iterations, Rician noise modelling, and accounting for gradient-non-linearity-induced b-matrix deviations.

For microstructural parameter estimation, NODDI-Bingham model was fitted to DWI data using a Bayesian inference framework with Markov chain Monte Carlo sampling.^35^ A burn-in of 5000 iterations was followed by 25 000 iterations with every 25th sample retained, yielding voxel-wise neurite density index (NDI), isotropic compartment fraction (ISO) and orientation dispersion index (ODI). Mapping of T_2_, T_1_ρ and T_2_ρ relaxation time constants was preceded by 3D rigid-body motion correction of all volumes to the first scan of each sequence (mutual information cost function with trilinear interpolation, followed by a refinement sinc interpolation step). Voxel-wise signal intensities were fitted with a two-parameter non-linear exponential decay model, estimating the equilibrium signal and the relaxation time via non-linear optimization (Nelder–Mead simplex).

All processing outputs were visually evaluated by a trained operator (P.F.), with quality control including also root-mean-squared voxel displacement in DWI, rs-fMRI, T_2_, T_1_ρ and T_2_ρ data (exclusion threshold of 2 mm) and eddy quality control metrics (mean absolute residuals, outlier slices) (exclusion threshold of 3 standard deviations of the cohort mean).

### Image parcellation and Bayesian graph-regularized probabilistic principal component analysis

Voxel-wise microstructural maps (T_1_, T_2_, T_1_ρ and T_2_ρ relaxation maps, ISO, ODI, NDI) were projected onto subject-specific mid-thickness cortical surfaces, constrained to the cortical ribbon defined by the HCP minimal preprocessing pipeline. Sampling along the cortical normal was performed with interpolation across the thickness dimension, weighting voxel contributions according to the proportion of grey matter intersected to minimize partial-volume contamination, followed by MSMAll-based alignment.^36^ Cortical parcellation was based on the multimodal HCP atlas,^37^ yielding 180 parcels per hemisphere. Subcortical grey-matter segmentation combined multiple probabilistic atlases covering all major structures: probabilistic structural cerebellar atlas,^38^ Oxford-GSK-Imanova connectivity striatal atlas,^39^ Oxford thalamic connectivity atlas,^40^ Hypothalamus atlas,^41^ ATAG atlas^42^ and ARAS atlas.^43^ In addition, amygdala nuclei and hippocampal subfields were delineated using the high-resolution FreeSurfer automatic segmentation framework.^44,45^ White-matter pathways were estimated utilizing probabilistic tractography employing subject-specific fibre orientations constrained with XTRACT standard anatomical priors to reconstruct major association, commissural and projection tracts.^46^ All atlas-derived masks were non-linearly warped from standard space to the individual T_1_-weighted anatomical image and subsequently rigidly aligned to the diffusion/quantitative mapping space in a single resampling step (trilinear interpolation). Regional values were obtained as weighted averages (the ratio of the voxel-wise product of intensity and probability to the sum of probabilities), thereby scaling each voxel’s contribution by its probabilistic membership in the respective parcel.

Remaining gaps in the dataset (T_2_ρ acquisition missing in two subjects) were filled by parcel-wise median imputation, features were winsorized at 0.5th and 99.5th percentile and *z*-scored. To encode anatomical structure, parcel groupings based on larger partition assignments (see Supplementary Table 1) defined a sparse binary adjacency matrix, from which a symmetric normalized graph Laplacian was derived and used to regularize the spatial smoothness of subsequent loading vectors. A Bayesian graph-regularized probabilistic principal component analysis (PPCA) model was then fitted separately for each MRI metric (for full model description including its implementation in Stan, see Supplementary Methods).

Latent dimensionality was selected across a candidate grid (*k* = 3, 5, 8, 11, 15) using 5-fold cross-validation, computing the coefficient of determination (*R*^2^) on held-out folds. The final *k* was determined via one-standard-error rule (smallest *k* with mean *R*^2^ within one standard error of the maximum), with preference for biologically interpretable solutions given the sample size and metric number (*k* = 8 or 11) if within the acceptable set corrected for marginal *R*^2^ gain (<0.01 threshold). Over-selection at the grid maximum was avoided if its *R*^2^ improvement over the previous candidate was <0.02. Stability across cross-validation folds was assessed using loading cosine similarity after orthogonal Procrustes alignment^47^ and principal angles between loading subspaces.

PPCA components were ranked by complete-case cohort sample variance, and the number of retained components *m* was determined using a label-agnostic approach—Marchenko–Pastur noise-floor criterion constrained to an interpretable range [3, 6], with broken-stick calculated for a sensitivity evaluation. The lower limit was set to match the number of considered cognitive measures (*Memory* and *Executive function* latent factors, CFQ) to ensure modality balance. The upper bound (twice the lower threshold) was selected to limit the discriminant below the Hotelling *T*^2^ feasibility bound, thereby preventing ill-conditioning and loss of power.

### Statistical analyses

Group comparability for demographic and clinical parameters was examined using bias-corrected and accelerated bootstrap 90% confidence intervals (BCa CI) (10 000 stratified resamples). Median differences were calculated for continuous variables, and differences in proportions for sex distribution. Standardized mean differences (SMDs) were used to quantify effect sizes.

### Aim 1: Group comparison over brain–cognition axis

Joint feature set comprising three cognitive and the top *m* MRI-PPCA scores was residualized for age (linear) and sex and mean-centred on the complete-case cohort. Group separation (Long COVID versus HC) was tested via Hotelling’s *T*^2^ with ridge-regularized pooled covariance (*λ* = 10^−4^) and 10 000 permutations. The observed squared Mahalanobis distance *D*^2^ was orthogonally decomposed via Cholesky whitening to attribute additive per-variable contributions of separation (aggregated into cognition versus MRI-PPCA blocks; and further by MRI metrics). Subject-level brain–cognition axis scores were obtained as the projection onto the regularized Mahalanobis (linear discriminant analysis) direction.

As a robustness check, the main Hotelling’s *T*^2^ test was repeated for m_MRI-PPCA_ = 3:10.

### Aim 2: Anatomical mapping

PPCA portions of *w* were mapped to parcels by multiplying component weights with the original Bayesian-PPCA loadings per metric. For metrics contributing with more than one MRI-PPCA components, parcel-level contribution sum was calculated, yielding a single MRI metric-specific parcel map. Top parcels were defined as the smallest subset explaining ≥50% of the L1 norm of the absolute weights.

### Aim 3: Relation of brain–cognition axis to symptom severity and domains

Associations between brain–cognition axis scores and Newcastle symptom dimensions (respiratory complaints, systemic symptoms, neurological/sensory–autonomic features) as well as the overall Newcastle score were examined using linear regression adjusted for age and sex. For each model, the strength of the relation was summarized by the regression coefficient and partial *R*^2^, with significance assessed via permutation testing with 10 000 covariate-preserving randomizations. Benjamini–Hochberg false discovery rate (FDR) control was applied across the four outcomes. Potential deviations from linearity of symptom–axis relations were assessed utilizing generalized additive models, with penalized splines estimated by restricted maximum likelihood.

## Results

The enrolment and exclusion process is summarized in the Supplementary Fig. 1. Most exclusion arose from the withdrawal of participants and incomplete MRI acquisition (missing >1 protocol). In the complete-case cohort utilized for the delineation of the brain–cognition axis (HC = 32; Long COVID = 77, see Table 1), the Long COVID group contained a higher proportion of women (64% versus 40% in HC; Δ ≈ +24 pp) and was modestly older (median +5.9 years; 90% BCa CI +2.3 to +9.1). Time since acute infection was comparable between groups (≈26 weeks; Δ ∼ 0). Hospitalization during the acute phase of COVID-19 infection occurred in 9% of the Long COVID group and in none of the controls. As expected, symptom burden was higher in Long COVID: Newcastle raw totals and domain scores (respiratory, systemic, neurological/sensory–autonomic) were all elevated.

**Table 1:** Cohort characteristics at enrolment

	Healthy controls	Long COVID	Δ inter-group [90% BCa CI]	SMD	*P*-value (Bonf)	Data avail.
Participants (complete-case cohort) [*n*]	32	77				
Sex (female/male) [*n* (% female)]	13/19 (40%)	49/28 (64%)	24% [5, 39]	0.47	0.30	32/77
Age [years]	40.8 [30.1, 56.6]	46.7 [32.5, 61.2]	5.9 [2.3, 9.1]	0.49	0.12	32/77
Time since acute infection [weeks]	25.9 [14.7, 35.0]	25.9 [15.5, 46.6]	0.0 [−3.9, 1.1]	0.27	1.00	32/69
History of hospitalization [*n* (%)]	0	7 (9%)	9% [5, 17]	0.44	0.86	32/77
Newcastle score [raw sum]	0.0 [0.0, 1.0]	5.0 [2.8, 10.0]	5.0 [5.0, 7.0]	2.40	<0.01*	28/69
Respiratory complaints [*z*-score]	−0.72 [−0.72, −0.70]	0.29 [−0.34, 1.23]	1.01 [0.82, 1.16]	1.93	<0.01*	28/69
Systemic symptoms [*z*-score]	−0.82 [−0.82, −0.61]	0.36 [−0.43, 1.14]	1.18 [1.01, 1.40]	2.25	<0.01*	28/69
Neurological/sensory–autonomic features [*z*-score]	−0.68 [−0.68, −0.28]	0.19 [−0.34, 1.16]	0.87 [0.75, 1.09]	1.78	<0.01*	28/69
CFQ [raw score]	31.0 [16.6, 42.9]	35.0 [20.0, 54.0]	4.0 [−1.5, 7.0]	0.37	0.54	32/77
Memory CFA latent factor [*z*-score]	0.61 [−0.73, 1.18]	−0.18 [−1.29, 0.95]	−0.79 [−1.05, −0.43]	0.77	<0.01*	32/77
Executive functions/attention CFA latent factor [*z*-score]	0.58 [−0.50, 1.21]	−0.25 [−1.33, 0.84]	−0.83 [−1.16, −0.54]	0.76	<0.01*	32/77

Values presented as median [10th–90th percentile] unless stated otherwise. Inter-group Δ is the median difference for continuous measures and the percentage-point difference for categorical measures between participants with Long COVID and healthy controls; negative Δ indicates lower values in Long COVID, provided 90% bias-corrected and accelerated bootstrap confidence intervals. SMD is absolute standardized mean difference using the pooled standard deviation. *P*-values denote conventional between-group statistical comparisons (Welch’s *t*-test for continuous variables and χ^2^ test for categorical variables), with Bonferroni correction for multiple comparisons; statistically significant differences marked with asterisk [*]. Data availability lists the number of participants with non-missing datapoints (healthy controls/Long COVID). Higher *z*-scores indicate worse symptoms (Newcastle subscores).

Abbreviations: BCa CI, bias-corrected accelerated bootstrap confidence intervals; Bonf, Bonferroni correction for multiple comparisons; CFA, confirmatory factor analysis; CFQ, Cognitive Failures Questionnaire; SMD, standardized mean difference.

**Figure 1:**
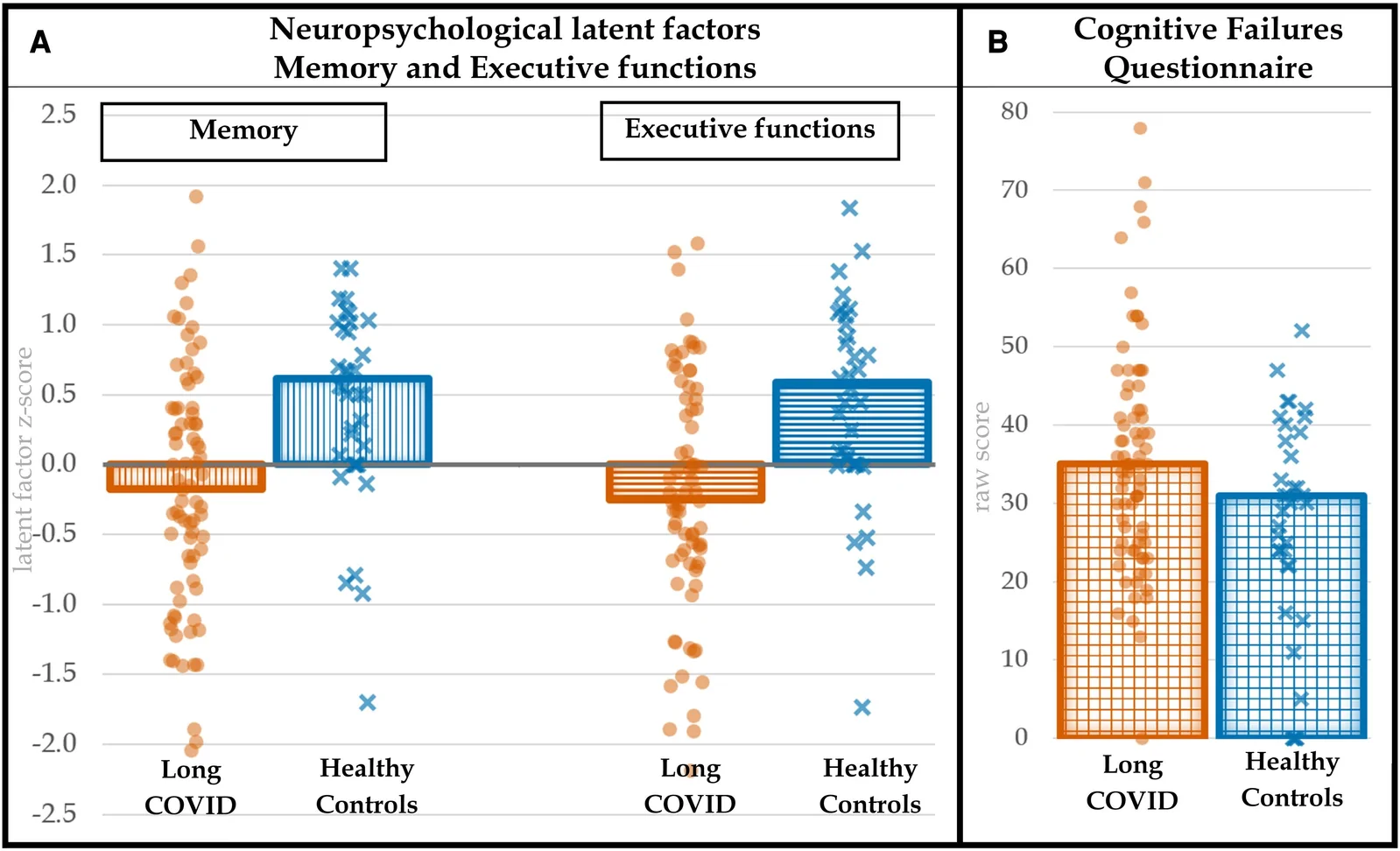
**Distributions of cognitive measures in participants with long COVID and healthy controls.** Each dot represents one participant (Long COVID = red; Healthy Controls = green), with jitter along the *x-*axis for better legibility. Boxes correspond to group medians. No inferential testing presented. (**A**) Latent factor scores for Memory and Executive functions/Attention (*z*-standardized so that higher = better performance). Healthy control medians tend to sit above Long COVID medians, but there is broad overlap and visible heterogeneity within each group. (**B**) Cognitive Failures Questionnaire (CFQ) raw scores (higher = more self-reported cognitive problems).

Posterior predictive checks for the Bayesian confirmatory factor model of cognitive performance indicated acceptable fit (PPP = 0.14). Sampling converged satisfactorily, with $\hat{R}$ across all parameters 0.9999–1.0006 (all <1.01) and effective sample sizes 5755–18 740 (median 10 225), supporting stable posterior summaries. Alternative model specifications with targeted cross-loadings [e.g. AVLT (recognition false-positive errors) – Rey–Osterrieth (copy trial)] yielded lower PPP values (0.09–0.11). Cognitive performance was lower in Long COVID, with both Memory and Executive function latent factors shifted downwards relative to controls (Δ ≈ −0.8 SD for each) (see Fig. 1). Moreover, CFQ scores were modestly higher (worse) in Long COVID (Δ ≈ +4 points).

All MRI metrics exhibited monotonic increase with rising dimensionality (mean *R*^2^ rising from *k* = 3 to *k* = 15); ranges by metric: T_1_ 0.58→0.69, T_2_ 0.28→0.42, T_1_ρ 0.38→0.53, T_2_ρ 0.37→0.51, ODI 0.18→0.29, ISO 0.29→0.42, NDI 0.37→0.54. The pre-specified selection rules yielded the following numbers of components: T_1_ρ *k* = 15, T_2_ρ *k* = 11, T_1_  *k* = 11, T_2_  *k* = 15, NDI *k* = 15, ISO *k* = 15, ODI *k* = 11. Where *k* = 15 was selected, the marginal gain over *k* = 11 satisfied the pre-specified guard (Δ*R*^2^ ≈ 0.02–0.03). Loading cosine stability across metrics ranged 0.88–0.97 and mean principal angles 16°–25°, demonstrating acceptable consistency of feature contributions and fold-wise variability. The per-metric embeddings were concatenated into a joint MRI view, for which global dimensionality was set by the Marchenko–Pastur noise-floor criterion to *m* = 5 (cumulative variance ≈ 0.295; *K*_all_ = 93; aspect ratio *q* ≈ 4.06). Nonetheless, the unsupervised broken-stick rule (raw) favoured substantially more components (*k* = 41, cumulative variance ≈ 0.770), which we judged over-parametrized given the *K*_all_ and high variable-to-subject ratio; hence, we retained *m* = 5 per primary criterion contained within the pre-selected interpretability range [3–6] and conducted sensitivity analyses across *m* = 3–10 at the subsequent level. The top five retained MRI-PPCA components are presented in the Supplementary Fig. 2.

### Aim 1: Group comparison over brain–cognition axis

Ridge-regularized Hotelling’s *T*^2^ implemented over the joint brain–cognition feature set revealed a statistically significant separation between participants with Long COVID and HC—at the primary setting *m* = 5, the test yielded *T*^2^ = 19.17, *D*^2^ = 0.848 (Mahalanobis distance *d*_M_ = 0.921) and a permutation *P* = 0.020. The orthogonal decomposition attributed 72.6% of *D*^2^ to cognition and 27.4% to MRI block. The largest single contribution came from the Memory latent factor (54.8% of *D*^2^) (see Table 2). Results were robust to both the use of norm-referenced neuropsychology latent factors (*T*^2^ = 21.87, *P* = 0.009) and the number of MRI-PPCA components (*m* = 3–8; *P* = 0.016–0.066), with attenuation at larger *m*, consistent with diminishing marginal gains when adding components (see Supplementary Table 2).

**Table 2:** Per-variable contributions to group separation on the brain–cognition axis

Block	Component	MRI-PPCA index	*D* ^2^ contribution	*D* ^2^ share	Weight *w*
Cognition	Memory latent factor	–	0.464	54.8%	−0.741
MRI	NDI—component 3	5	0.122	14.4%	−0.830
Cognition	CFQ	–	0.102	12.1%	0.029
MRI	ISO—component 14	3	0.056	6.6%	0.586
Cognition	Executive latent factor	–	0.049	5.7%	−0.363
MRI	ISO—component 4	2	0.043	5.1%	0.038
MRI	ISO—component 6	4	0.006	0.7%	0.464
MRI	T_2_ map—component 9	1	0.005	0.6%	0.075

Contributions are from the orthogonal (whitened) decomposition of *D*^2^, their shares sum to 1. ‘Weight *w*’ are coefficients from the ridge-regularized Mahalanobis/LDA direction. For MRI entries, names show metric and the component within that metric; MRI-PPCA index denotes the position of the component in the variance-ranked MRI-PPCA feature set (distinct from the within-metric component number). Contributions (*D*^2^ shares) are invariant to sign.

Abbreviations: CFQ, Cognitive Failures Questionnaire. *D*^2^, squared Mahalanobis distance; ISO, isotropic fraction (NODDI isotropic compartment); MRI-PPCA, probabilistic principal component analysis of magnetic resonance imaging inputs; NDI, neurite density index (NODDI intra-neurite fraction); T_2_ map, quantitative transverse relaxation map.

### Aim 2: Anatomical mapping

MRI contribution to the brain–cognition axis was dominated by isotropic fraction (ISO) components, with unit-norm normalized weights for ISO–component 14 (+0.421) and ISO–component 6 (+0.334), and a smaller ISO–component 4 (+0.027). In contrast, the NDI component 3 carried an opposite sign (−0.597), indicating that higher NDI estimates were associated with lower axis scores. A single T_2_-map component 9 made a modest positive contribution (+0.054). Nonetheless, the cohort-wise single-modal summary in Supplementary Table 3 shows no systematic univariate patterns, with largely overlapping distributions of individual MRI metrics (Vargha–Delaney *A* ≈ 0.50 ± 0.05).^48^

**Figure 2:**
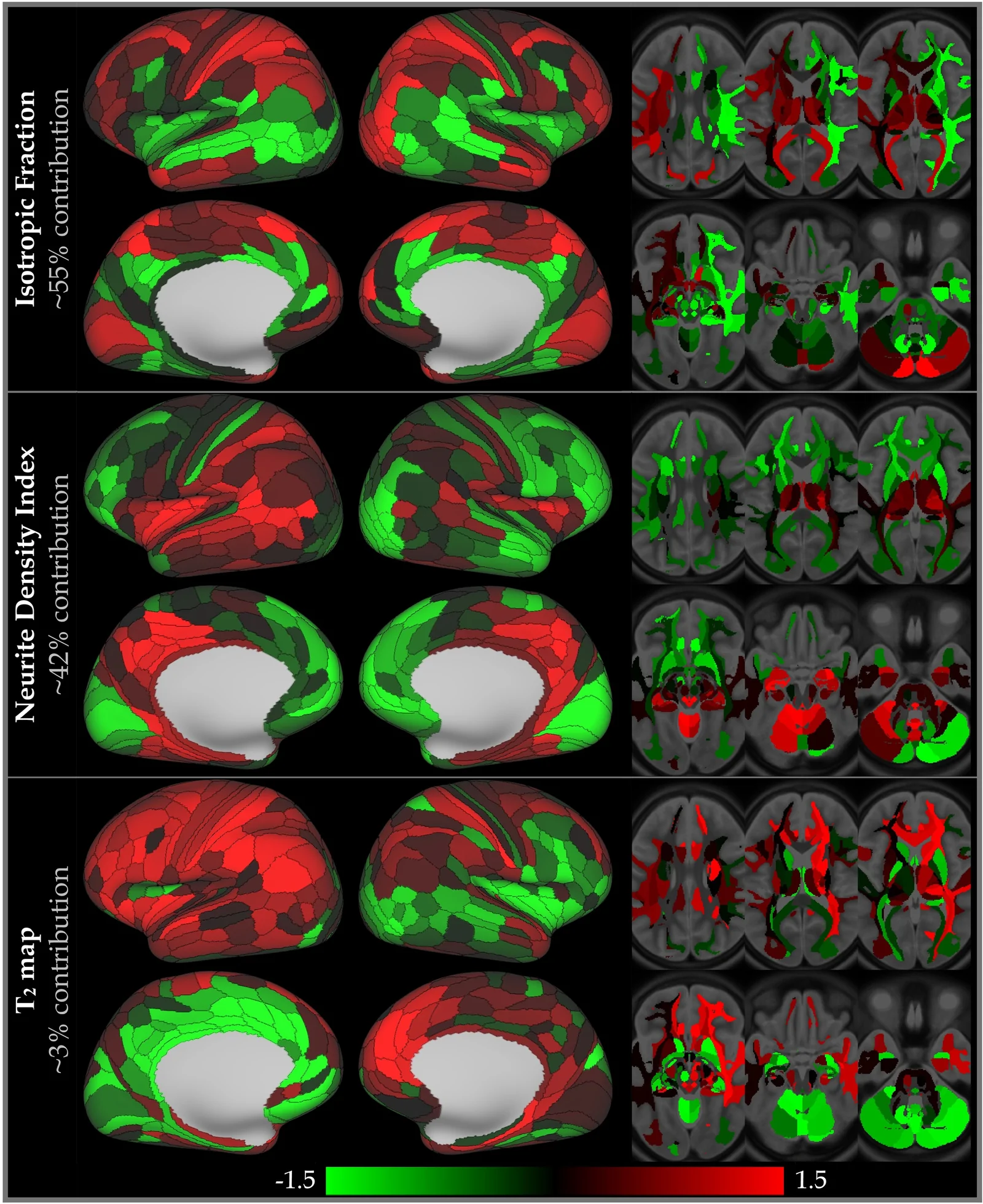
**Anatomical mapping of MRI contributions to the brain–cognition axis.** Parcel-wise maps show the projection of the discriminant weights back to anatomy. Left panels show lateral and medial cortical surfaces; right panels show subcortical axial slices in Montreal Neurological Space [*z* = 28, 16, 4, −8, −20, −32]. Images follow neurological convention (right = right). Colours encode within-metric normalized parcel weights (scale bar −1.5 to +1.5), with red indicating more positive (Long COVID-like) and green more negative (closer to healthy controls) association with the brain–cognition axis. NODDI isotropic fraction components jointly carried ∼55% of the MRI block’s absolute discriminant weight (‖*w*‖_1_), followed by NDI (∼42%); the T_2_ map component contributed only a small share (the values indicate each metric’s share of the MRI block contribution to the brain–cognition axis, not the total including cognition). Prominent spatial patterns involve fronto-parietal/dorsal-attention and cingulo-opercular–salience networks with thalamo-striatal and cerebellar circuits. See Supplementary Table 4 for a full list.

In the projection of the discriminant weights back to parcel level (see Fig. 2 and Supplementary Table 4), ISO-driven effects clustered in the dorsal attention/fronto-parietal control systems (superior parietal/IPS, dorsolateral prefrontal, inferior parietal) and the cingulo-opercular/salience network (anterior/posterior cingulate, mid-cingulate/paracentral, insula). The subcortical weights covered thalamo-striatal territories and cerebellum. The NDI map aligned with default-mode/limbic circuits, centred on posterior cingulate/precuneus, hippocampal formation and amygdala, compatible with meso-corticolimbic integration. Patterns were predominantly bilateral across the NODDI metrics. The T_2_-map component covered executive–salience hubs (dorsolateral prefrontal, anterior cingulate, insula) with left-hemisphere predominance, but also together with premotor/sensorimotor cortex, thalamus, striatum, and cerebellar regions, consistent with cortico-striato-thalamo-cerebellar loops supporting attention and action selection.

### Aim 3: Relation of brain–cognition axis to symptom severity and domains

**Figure 3:**
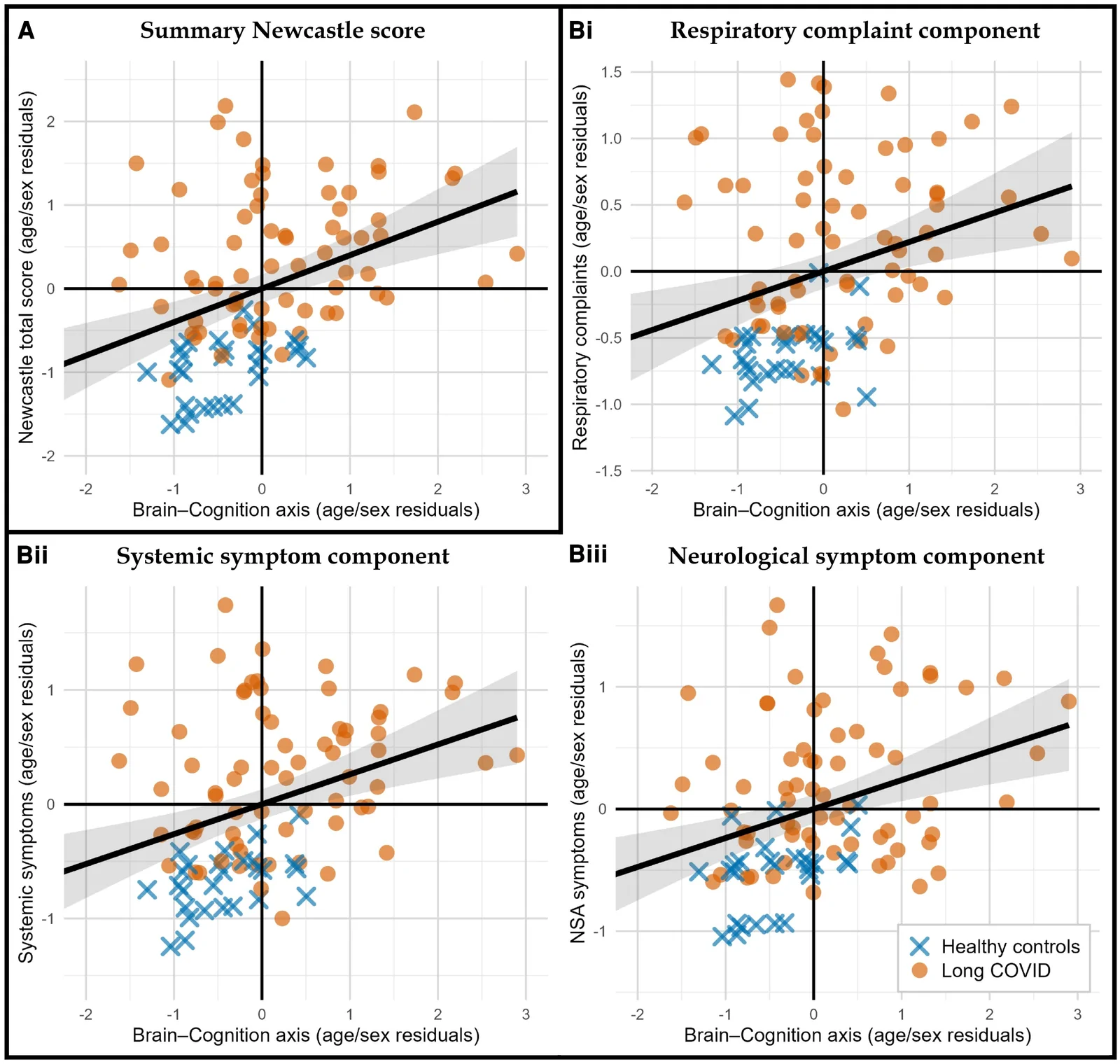
**Brain–cognition axis versus Newcastle symptom burden.** Each panel shows a partial-regression scatterplot after residualizing both variables for age and sex (orange points = participants with Long COVID; blue crosses = healthy controls; solid line = linear fit; shaded band = 95% confidence interval; grey crosshairs mark zero). Axes standardized to the residual scale (*x*: brain–cognition axis; *y*: symptom dimension). (**A)** Total Newcastle score. (**Bi–Biii**) Positive associations for individual subcomponents: Respiratory complaints, Systemic symptoms and Neurological/sensory–autonomic (NSA) features. *N* = 97 (69 Long COVID, 28 healthy controls) in each panel. Age- and sex-adjusted associations: total Newcastle score, partial *R*^2^ = 0.178, *P*_FDR_ < 0.001; respiratory, partial *R*^2^ = 0.102, *P*_FDR_ = 0.002; systemic, partial *R*^2^ = 0.137, *P*_FDR_ < 0.001; neurological/sensory–autonomic, partial *R*^2^ = 0.134, *P*_FDR_ = 0.001. *P*_FDR_ values are permutation *P*-values adjusted across the four outcomes using Benjamini–Hochberg FDR procedure.

Higher brain–cognition axis scores were associated with greater symptom burden across all Newcastle domains (respiratory, systemic, neurological/sensory–autonomic) and with the overall Newcastle score as well, with FDR-corrected significance in every case (*q* ≤ 0.01) (see Fig. 3 and Supplementary Table 5). Effect size analysis yielded partial *R*^2^ ≈ 0.10–0.18, strongest for the overall Newcastle score. Generalized additive models showed no evidence of non-linearity (edf ≈ 1.00 throughout).

## Discussion

The recent surge in the research into the protracted impacts of SARS-CoV-2 has so far largely failed to capitalize on the advances in microstructural MRI. While standard univariate comparisons of individual imaging or neuropsychology measures alone often missed group differences,^18-20^ the presented study interrogated subtle neurobiological effects of Long COVID via a single discriminant brain–cognition axis. Implementing a high-dimensional multimodal MRI pipeline combined with an extensive neuropsychological battery, this integrated analytical approach detected a coherent shared variance pattern linking microstructural alterations and cognition with significant group separation. Consistent with the ‘brain fog’ phenomenon, lower objective cognitive performance in the Long COVID patients was combined with distributed microstructural contributions spanning fronto-parietal, limbic and cortico-subcortical networks on MRI metrics sensitive to neuroinflammatory processes.^25^ Moreover, higher brain–cognition axis scores were associated with greater symptom burden without clear predominance of respiratory, systemic or neurocognitive domains (see Fig. 3).

### The brain–cognition axis as an integrative framework

Central to the presented study is the concept of the brain–cognition axis—a discriminant latent dimension integrating multimodal MRI and cognitive data into a single analytic framework. This construct reframes multimodal data not as parallel and separate domains, but as interdependent signals within a shared latent process. Given its multifaceted, diffuse nature, Long COVID is unlikely to produce focal lesions or isolated deficits, hence making traditional univariate analyses insufficiently sensitive due to their treatment of noise and signal as independent across the considered modalities as evident in the previous body of literature^18-20^ but also in the presented dataset (see Supplementary Table 3). However, the joint modelling of the brain–cognition axis capitalized on their covariance structure and was able to extract a coherent multivariate pattern maximizing group discrimination. Hence, this brain–cognition axis can be seen as a multimodal biomarker where each individual’s position on the axis reflects a weighted combination of their cognitive performance and microstructural brain features with high relevance to symptom severity scales (see Fig. 3). Notably, no clear predominance of respiratory, systemic or neurological symptom domains was detected. Furthermore, all participants with subjective complaints underwent specialized internal medicine evaluation without clear evidence of peripheral organ pathology, which could account for reported complaints. Hence, such clinical manifestations may reflect a shared underlying process rather than symptom-specific mechanisms, positioning the brain–cognition axis as a marker of the overall severity of the Long COVID condition.

Crucially, the axis allowed for direct quantification of the relative contributions of cognitive performance and brain microstructure to group separation. While cognitive factors accounted for ∼73% of the discriminant variance, imaging components provided complementary information indicating a strong anchor of the cognitive phenotype in distributed microstructural alterations (see Fig. 2). The MRI-derived component, though accounting for a smaller proportion of discriminant variance, was indispensable for achieving full group separation—indicating that cognition and brain structure carry partially non-overlapping information. Hence, purely neuropsychological assessments would underestimate the pathophysiological footprint of Long COVID. Careful evaluation of robustness, including analyses of dimensionality sensitivity and permutation testing, confirmed the stability of the proposed brain–cognition axis and its discriminant abilities across a reasonable range of modelling choices. Nonetheless, while joint modelling of multimodal data shows future promise in disentangling complex physiological and pathophysiological processes, the generalizability of this concept is yet to be evaluated in other, external Long COVID cohorts, ideally in combination with the dataset shared as a part of this paper.

### Potential mechanistic links: neuroinflammation and microvascular dysfunction

The discriminant weighting of MRI features (see Fig. 2 and Supplementary Table 4) offers insight into the underlying neurobiological alterations present in Long COVID. We observed that diffusion MRI features—particularly those reflecting extracellular free water (isotropic diffusion fraction)—were heavily weighted on the Long COVID side of the brain–cognition axis. In practical terms, higher free-water content in tissue could indicate diffuse inflammatory oedema, glial activation or even blood-brain barrier leak in affected individuals.^25^ In parallel, NDI carried substantial weight with an opposite sign, meaning Long COVID patients tended to have lower NDI in key regions compared to controls. In grey matter regions, this points to subtle microstructural degeneration or sparser neurite architecture—specifically deterioration of dendritic complexity and other unmyelinated features in the cortical regions.^49^ These affections were mostly distributed across larger networks spanning the dorsal attention and salience systems but also the limbic system and several cortico-subcortical circuits. This spatial pattern is highly concordant with the diffuse subjective complaints, executive and attentional impairment and memory dysfunction detected in our cohort (see Fig. 1 and Table 1), but also in prior reports,^17,50,51^ underscoring the network-wide characteristics of this condition.

By contrast, quantitative relaxometry measures (T_1_, T_2_, T_1_ρ, T_2_ρ), which are sensitive to myelin, iron, and biochemical microenvironment, failed to yield more substantial contributions to the discriminant axis. Transverse relaxation time provided a very small positive weight (only 3% of the MRI block discriminant weight), yet overall, the relaxometry changes were not dominant. This relative insensitivity suggests that the microstructural impact of Long COVID is unlikely to be centred upon large-scale demyelination or iron deposition in individuals with the history of mild acute infection. This pattern is dissimilar to neurodegeneration or primary neuroinflammatory conditions where rotating-frame relaxation metrics dominated,^52-54^ but reminiscent of cerebral findings in systemic inflammation.^55^

Mechanistically, one possible interpretation postulates chronic, low-grade, diffuse neuroinflammatory changes more so than extensive demyelination or tissue loss—a conclusion well aligned with current hypotheses of immune-driven pathology in Long COVID^4-6^. Secondary contributions from microvascular involvement and blood-brain barrier disruption, without clear evidence of an initial acute destructive event, are also plausible pathophysiological pathways,^7^ supported by previously reported regional hypoperfusion.^14,15^ Nonetheless, these inferences, while biologically plausible, remain tentative given the indirect nature of MRI markers. Quantitative MRI offers generally high sensitivity to a wide spectrum of overlapping biological processes, but often only modest specificity to underlying pathophysiology. No single MRI metric may be considered an exclusive proxy for any individual biological feature. It is the strength of the presented technical approach, however, that such inferences can be drawn at all. Only by combining multiple features did consistent patterns appear where traditional single-modality analyses of similar cohorts with mild acute infection course failed^18-20^ and positive findings were largely confined to severe acute infection cases.^16^ All in all, the convergence of diffusion abnormalities in clinically relevant networks with measurable cognitive deficits strengthens the proposed interpretation of persistent immune-mediated and vascular processes as key contributors to the pathophysiology of Long COVID.

### Limitations and considerations

Notwithstanding its strengths, our study has several important limitations. Although our cohort is one of the largest neuroimaging studies of Long COVID to date, the high dimensionality of our feature space necessitated aggressive dimension reduction and regularization. Although essential to avoid overfitting, the undertaken steps may have reduced model stability or could obscure subtler effects. This concern is further accentuated by the cross-sectional design demonstrating group separation at one time point in a cohort deliberately selected to exclude severe acute infection course. While a sensible choice due to the high risk of confounding by sequelae of critical illness, our findings may not extend to patients who experienced intensive care and mechanical ventilation who may exhibit more complex brain changes.

Secondly, the MRI pipeline is highly specialized and technically demanding, requiring advanced acquisition protocols, specific hardware and considerable computational resources. The analytical framework involving multivariate modelling, regularized dimensionality reduction and feature integration is inherently complex and introduces multiple modelling decisions (e.g. dimensionality reduction thresholds, regularization parameters, parcellation schemes). Although we undertook extensive cross-validation, sensitivity analyses and projection back to biologically meaningful networks and associations with clinical symptom severity, thereby supporting the validity of the concept, this approach is currently impractical for routine clinical implementation.

Lastly, the causality and inferred pathophysiological mechanism remain speculative. Mere association between brain alterations, cognitive performance and symptom burden cannot exclude a higher-order process driving all the domains in parallel. Further potentially relevant dimensions such as medical history, education, mood, sleep quality, pain or systemic inflammation markers which may mediate brain alterations were not incorporated into the analysis and may contribute to or mediate the observed alterations.

## Conclusion

Our study provides convergent evidence of Long COVID as a complex neurological entity involving subtle diffuse alterations to cerebral microstructure coupled with cognitive impairment. The multimodal biomarker engineered from complex quantitative MRI and neuropsychological data—the latent brain–cognition axis—not only separated patients from controls but also tracked the Long COVID symptom burden. The prominence of neuroinflammation-sensitive MRI protocols highlights immune and vascular contributions rather than overt neurodegeneration. Longitudinal validation and protocol extension by further biomarker dimensions will be essential to determine prognostic value and facilitate wider adoption.

## Supplementary Material

fcag344_Supplementary_Data

## Data Availability

Parcellated MRI dataset of derived microstructural metrics (T_1_, T_2_, T_1_ρ and T_2_ρ relaxation maps, ISO, ODI, NDI) is available as Supplementary material. Raw unprocessed data are available from the corresponding author upon reasonable request. MRI data processing utilized HCP pipeline available at https://github.com/Washington-University/HCPpipelines. Statistical analyses were performed using standard statistical software and publicly available packages. No custom methodological software was developed for this study.
