# Supplementary material for: Latent brain–cognition dimension indicative of neuroinflammatory basis of Long COVID
Source: Brain Commun. 2026 Sep 19;8(5):fcag344. doi: 10.1093/braincomms/fcag344 (PMC13599023; doi:10.1093/braincomms/fcag344)
Supplement: Supplementary file 1 [file fcag344_supplementary_data.zip › Supplementary_material.docx]

# Supplementary Methods

**Spatial graph construction and Bayesian graph-regularised PPCA**

**Spatial adjacency graph and Graph Laplacian**

The anatomical pooling of parcels was encoded as an adjacency graph derived from larger anatomical partitions (Supplementary Table 1). Parcels belonging to the same larger *Partition Segment* formed fully connected cliques (encoded as 1), while edges to different partition parcels and self-connections were set to 0.

This sparse binary adjacency matrix *A* was utilised to compute a symmetric normalised graph Laplacian

Let

$$D_{ii}=\sum_{j} A_{ij}$$

denote the node degree matrix. The Laplacian is then defined as a positive semi-definite matrix

$$L=I-D^{-1/2}AD^{-1/2}$$

where *I* is the identity matrix.

**Bayesian Graph-Regularised Probabilistic PCA**

The parcel-wise data matrix for each MRI metric was encoded as $X\in\mathbb{R}^{n\times p}$ with *n* subjects and *p* parcels and modelled using a probabilistic PCA formulation $X=ZW^{\top}+\varepsilon$ where:

- $Z\in\mathbb{R}^{n\times k}$stands for latent subject scores
- $W\in\mathbb{R}^{p\times k}$ stands for parcel loading vectors
- *ε* denotes independent Gaussian residual noise, with $\varepsilon\sim N(0,\sigma^{2})$

*k* is the number of latent factors (selected separately from candidate grid using five-fold cross validation for each MRI metric – see Methods in the main text).

Spatial regularisation utilising the previously described Laplacian matrix *L* was implemented by assigning a multivariate Gaussian prior with precision matrix $\left( \tau_{j}I + \lambda L \right)$ to each loading vector *W_.j_*

$$W_{\cdot j}\sim N\text{ }\left( 0,(\tau_{j}I+\lambda L)^{-1} \right)$$

where *τ_j_* is a factor-specific shrinkage parameter and *λ* encodes the extent of Laplacian smoothing. This formulation thus promotes spatial smoothness of loading vectors within same anatomical *Partition Segments.*

**Prior specification**

- Latent subject scores	$Z_{ij}\sim N(0,1)$
- Residual scale	$\sigma\sim t_{3}(0,1)^{+}$
- Factor-specific shrinkage	$\tau_{j}\sim t_{3}(0,1)^{+}$
- Laplacian penalty parameter	$\lambda\sim t_{3}(0,1)^{+}$

**Posterior inference**

Automatic variational inference (ADVI) as implemented in cmdstanr was utilised. 20,000 optimisation iterations were run for each model, and 1,000 posterior draws were sampled from the approximation. Posterior means of *Z* and *W* were then provided as point estimates for subsequent steps.

**Full Stan implementation**

// n: number of subjects

// p: number of retained parcels/features for the current MRI metric

// k: number of latent PPCA components

// X: preprocessed subject-by-parcel data matrix

// L: symmetric normalised graph Laplacian encoding anatomical parcel adjacency

// Z: subject-level latent component scores

// W: parcel loading matrix

// sigma: residual standard deviation

// tau: component-specific loading shrinkage

// lambda: strength of graph-Laplacian spatial regularisation

**data {**

int<lower=1> n;

int<lower=1> p;

int<lower=1> k;

matrix[n, p] X;

matrix[p, p] L;

}

**parameters {**

matrix[n, k] Z;

matrix[p, k] W;

real<lower=0> sigma;

vector<lower=0>[k] tau;

real<lower=0> lambda;

}

**model {**

// Latent subject scores

to_vector(Z) ~ normal(0, 1);

// Weakly informative half-Student-t priors

sigma ~ student_t(3, 0, 1);

tau	~ student_t(3, 0, 1);

lambda ~ student_t(3, 0, 1);

// Graph-regularised loading prior:

for (j in 1:k) {

matrix[p, p] Q;

Q = add_diag(lambda * L, tau[j]);

col(W, j) ~ multi_normal_prec(rep_vector(0, p), Q);

}

// Observation model

to_vector(X) ~ normal(to_vector(Z * W'), sigma);

}

# Supplementary Tables

**Supplementary Table 1: Overarching partition segments** **used to define parcel groupings for the graph adjacency matrix** in the Bayesian graph regularised probabilistic principal component analysis. Within each segment, all parcels were treated as mutually connected (clique structure) when constructing the adjacency matrix.

|  | Partition Segment | Parcel no. | Category | Source Atlas |
| --- | --- | --- | --- | --- |
| 1 | Primary Visual Cortex | 2 | **Visual/ Occipital Cortex** | Human Connectome Project Multimodal Parcellation Atlas (1) |
| 2 | MT+ Complex and Neighbouring Visual Areas | 18 |  |  |
| 3 | Dorsal Stream Visual Cortex | 12 |  |  |
| 4 | Early Visual Cortex | 6 |  |  |
| 5 | Ventral Stream Visual Cortex | 14 |  |  |
| 6 | Somatosensory and Motor Cortex | 10 | **Sensori-motor Cortex** |  |
| 7 | Premotor Cortex | 14 |  |  |
| 8 | Posterior Opercular Cortex | 12 |  |  |
| 9 | Paracentral Lobular and Mid Cingulate Cortex | 16 |  |  |
| 10 | Early Auditory Cortex | 10 | **Auditory Cortex** |  |
| 11 | Auditory Association Cortex | 16 |  |  |
| 12 | Insular and Frontal Opercular Cortex | 26 |  |  |
| 13 | Medial Temporal Cortex | 14 | **Temporal Cortex** |  |
| 14 | Lateral Temporal Cortex | 18 |  |  |
| 15 | Temporal-Parietal-Occipital Junction | 10 | **Parietal Cortex** |  |
| 16 | Superior Parietal and IPS | 20 |  |  |
| 17 | Inferior Parietal Cortex | 20 |  |  |
| 18 | Posterior Cingulate Cortex | 28 |  |  |
| 19 | Anterior Cingulate and Medial Prefrontal Cortex | 30 | **Frontal Cortex** |  |
| 20 | Orbital and Polar Frontal Cortex | 22 |  |  |
| 21 | Inferior Frontal Cortex | 16 |  |  |
| 22 | Dorsolateral Prefrontal Cortex | 26 |  |  |
| 23 | Thalamus | 14 | **Dience-phalon** | Oxford Thalamic Connectivity Probability Atlas (2) |
| 24 | Hypothalamus | 4 |  | Hypothalamic atlas (3) |
| 25 | Striatum | 14 | **Basal Ganglia** | Oxford-Imanova Striatal Atlas (4) |
| 26 | Pallidum | 4 |  | ATAG for middle aged, elderly (5) |
| 27 | Basal Ganglia-Associated Nuclei | 4 |  |  |
| 28 | Brainstem – Monoaminergic and Arousal Nuclei | 3 | **Brainstem** | Harvard Ascending Arousal Network Atlas*)*(6) |
| 29 | Brainstem – Reticular and Sensorimotor Integration Nuclei | 7 |  |  |
| 30 | Amygdala | 8 | **Limbic System** | High-resolution atlas of amygdala  (7) |
| 31 | Hippocampus | 20 |  | In Vivo High-Resolution Atlas of the Hippocampus and Subfields (8) |
| 32 | Cerebellar Cortex | 27 | **Cerebellum** | Probabilistic structural cerebellar atlas  (9) |
| 33 | Cerebellar Deep Nuclei | 4 |  |  |
| 34 | Commissural Fibres | 4 | **White Matter** | XTRACT – Cross-Tract Probabilistic Tractography Protocols  (10) |
| 35 | Association Fibres | 20 |  |  |
| 36 | Projection Fibres | 10 |  |  |
| 37 | Limbic Fibres | 8 |  |  |

**Supplementary Table 2: Sensitivity of group separation (Long COVID vs healthy controls) to the number of MRI–PPCA components** (*m*). Provided separately for the main analysis considering raw neuropsychological scores and robustness analysis based on norm-referenced neuropsychological scores. Results from ridge-regularised Hotelling’s T^2^ T (λ=10^−4^); D^2^ is the squared Mahalanobis distance and d_M_=$\sqrt{D^{2}}$. p-values are from 10,000 label permutations.

| Number of MRI-PPCA components | Main analysis | | | | Robustness analysis | | | |
| --- | --- | --- | --- | --- | --- | --- | --- | --- |
|  | **T^2^** | **D^2^** | **d_M_** | **p value** | **T^2^** | **D^2^** | **d_M_** | **p value** |
| 3 | 16.260 | 0.719 | 0.848 | 0.016 | 19.303 | 0.854 | 0.924 | 0.005 |
| 4 | 16.402 | 0.726 | 0.852 | 0.025 | 19.322 | 0.855 | 0.925 | 0.009 |
| 5 | 19.166 | 0.848 | 0.921 | 0.020 | 21.869 | 0.967 | 0.984 | 0.009 |
| 6 | 19.489 | 0.862 | 0.929 | 0.034 | 22.040 | 0.975 | 0.987 | 0.014 |
| 7 | 19.933 | 0.882 | 0.939 | 0.052 | 22.520 | 0.996 | 0.998 | 0.024 |
| 8 | 20.541 | 0.909 | 0.953 | 0.066 | 23.120 | 1.023 | 1.011 | 0.037 |
| 9 | 20.780 | 0.919 | 0.959 | 0.095 | 23.468 | 1.038 | 1.019 | 0.051 |
| 10 | 20.782 | 0.919 | 0.959 | 0.148 | 23.481 | 1.039 | 1.019 | 0.083 |

**Supplementary Table 3: Descriptive single-modal summary of individual MRI metrics for Long COVID and healthy controls.** For each major partition segment (see Supplementary Table 1), median and 10^th^-90^th^ percentile within respective cohort is presented. Vargha-Delaney A index expresses the probability that a random Long-COVID value exceeds a random control value (A = 0.5 indicated complete overlap; A < 0.5 favours higher values in controls; A > 0.5 favours higher values in Long-COVID patients). ΔMedian is the percentual difference of cohort medians. No inferential statistics applied.

| Region | MRI metric | Long COVID Median [P10–P90] | Healthy controls Median [P10–P90] | Vargha–Delaney A  (Long COVID > HC) | ΔMedian [%] |
| --- | --- | --- | --- | --- | --- |
| Primary Visual Cortex | **T_1_ρ map [ms]** | 160.7 [155.7-167.1] | 162.2 [157.2-167.4] | 0.431 | -1.0% |
|  | **T_2_ρ map [ms]** | 77.1 [74.0-82.2] | 77.1 [72.7-82.5] | 0.470 | 0.0% |
|  | **T_1_ map [ms]** | 1276.2 [1229.1-1316.3] | 1275.8 [1229.3-1306.3] | 0.507 | 0.0% |
|  | **T_2_ map [ms]** | 98.9 [91.7-107.6] | 100.2 [90.1-109.1] | 0.490 | -1.2% |
|  | **NODDI NDI** | 0.412 [0.395-0.431] | 0.407 [0.390-0.433] | 0.591 | 1.3% |
|  | **NODDI ISO** | 0.014 [0.005-0.035] | 0.013 [0.006-0.032] | 0.495 | 5.1% |
|  | **NODDI ODI** | 0.608 [0.578-0.633] | 0.601 [0.581-0.627] | 0.544 | 1.1% |
| MT+ Complex and Neighbouring Visual Areas | **T_1_ρ map [ms]** | 156.1 [149.5-162.6] | 155.9 [150.4-163.4] | 0.484 | 0.1% |
|  | **T_2_ρ map [ms]** | 72.8 [68.9-77.8] | 72.4 [68.5-77.5] | 0.522 | 0.5% |
|  | **T_1_ map [ms]** | 1341.5 [1282.9-1393.8] | 1343.3 [1288.4-1394.6] | 0.476 | -0.1% |
|  | **T_2_ map [ms]** | 92.0 [85.1-101.2] | 92.0 [84.8-101.5] | 0.503 | 0.0% |
|  | **NODDI NDI** | 0.415 [0.393-0.436] | 0.412 [0.390-0.438] | 0.542 | 0.9% |
|  | **NODDI ISO** | 0.000 [0.000-0.004] | 0.000 [0.000-0.004] | 0.504 | 12.5% |
|  | **NODDI ODI** | 0.600 [0.548-0.652] | 0.599 [0.551-0.650] | 0.495 | 0.1% |
| Dorsal Stream Visual Cortex | **T_1_ρ map [ms]** | 162.0 [153.2-175.0] | 162.6 [153.0-175.7] | 0.485 | -0.4% |
|  | **T_2_ρ map [ms]** | 78.8 [72.1-87.8] | 78.4 [72.0-87.7] | 0.513 | 0.5% |
|  | **T_1_ map [ms]** | 1296.7 [1239.3-1349.0] | 1295.9 [1240.9-1344.0] | 0.505 | 0.1% |
|  | **T_2_ map [ms]** | 94.8 [87.0-106.3] | 94.0 [86.7-104.6] | 0.533 | 0.9% |
|  | **NODDI NDI** | 0.413 [0.389-0.439] | 0.413 [0.386-0.440] | 0.509 | 0.1% |
|  | **NODDI ISO** | 0.002 [0.000-0.015] | 0.002 [0.000-0.020] | 0.483 | -2.7% |
|  | **NODDI ODI** | 0.590 [0.545-0.631] | 0.590 [0.541-0.635] | 0.503 | 0.1% |
| Early Visual Cortex | **T_1_ρ map [ms]** | 156.6 [151.5-162.2] | 157.1 [152.3-163.5] | 0.446 | -0.3% |
|  | **T_2_ρ map [ms]** | 74.3 [71.2-78.2] | 74.5 [70.5-79.1] | 0.485 | -0.3% |
|  | **T_1_ map [ms]** | 1304.4 [1261.3-1345.9] | 1307.4 [1266.0-1343.6] | 0.488 | -0.2% |
|  | **T_2_ map [ms]** | 92.8 [88.0-99.2] | 93.4 [87.0-100.2] | 0.482 | -0.7% |
|  | **NODDI NDI** | 0.421 [0.403-0.438] | 0.415 [0.398-0.443] | 0.574 | 1.4% |
|  | **NODDI ISO** | 0.006 [0.001-0.022] | 0.007 [0.001-0.027] | 0.474 | -11.9% |
|  | **NODDI ODI** | 0.610 [0.584-0.633] | 0.609 [0.589-0.630] | 0.515 | 0.1% |
| Ventral Stream Visual Cortex | **T_1_ρ map [ms]** | 158.0 [152.2-164.6] | 157.9 [152.1-163.6] | 0.512 | 0.1% |
|  | **T_2_ρ map [ms]** | 72.9 [69.8-77.2] | 72.8 [69.4-76.8] | 0.503 | 0.1% |
|  | **T_1_ map [ms]** | 1332.5 [1282.8-1381.0] | 1330.7 [1280.2-1382.8] | 0.511 | 0.1% |
|  | **T_2_ map [ms]** | 88.7 [82.9-95.0] | 88.5 [82.0-94.6] | 0.522 | 0.2% |
|  | **NODDI NDI** | 0.428 [0.406-0.453] | 0.428 [0.405-0.455] | 0.497 | 0.0% |
|  | **NODDI ISO** | 0.003 [0.000-0.025] | 0.004 [0.000-0.027] | 0.486 | -2.2% |
|  | **NODDI ODI** | 0.603 [0.556-0.646] | 0.604 [0.563-0.647] | 0.480 | -0.2% |
| Somatosensory and Motor Cortex | **T_1_ρ map [ms]** | 172.3 [159.7-186.6] | 171.3 [160.6-188.0] | 0.515 | 0.6% |
|  | **T_2_ρ map [ms]** | 85.4 [77.9-96.7] | 85.4 [77.9-97.8] | 0.496 | 0.1% |
|  | **T_1_ map [ms]** | 1278.4 [1220.6-1346.1] | 1284.3 [1224.5-1344.8] | 0.478 | -0.5% |
|  | **T_2_ map [ms]** | 99.9 [91.7-113.6] | 99.2 [92.4-112.2] | 0.519 | 0.8% |
|  | **NODDI NDI** | 0.431 [0.398-0.471] | 0.429 [0.392-0.473] | 0.501 | 0.4% |
|  | **NODDI ISO** | 0.022 [0.004-0.085] | 0.023 [0.004-0.080] | 0.497 | -5.6% |
|  | **NODDI ODI** | 0.565 [0.457-0.609] | 0.563 [0.448-0.613] | 0.498 | 0.4% |
| Premotor Cortex | **T_1_ρ map [ms]** | 169.7 [159.1-181.9] | 168.6 [158.6-179.8] | 0.538 | 0.6% |
|  | **T_2_ρ map [ms]** | 81.1 [75.3-90.4] | 80.9 [74.8-89.6] | 0.516 | 0.2% |
|  | **T_1_ map [ms]** | 1347.1 [1294.7-1404.9] | 1353.6 [1300.4-1406.4] | 0.467 | -0.5% |
|  | **T_2_ map [ms]** | 98.2 [91.6-108.2] | 97.7 [91.0-107.7] | 0.520 | 0.6% |
|  | **NODDI NDI** | 0.426 [0.401-0.459] | 0.425 [0.400-0.460] | 0.512 | 0.3% |
|  | **NODDI ISO** | 0.007 [0.001-0.029] | 0.006 [0.001-0.026] | 0.533 | 16.7% |
|  | **NODDI ODI** | 0.551 [0.499-0.594] | 0.554 [0.502-0.600] | 0.480 | -0.5% |
| Posterior Opercular Cortex | **T_1_ρ map [ms]** | 170.5 [160.9-183.8] | 169.5 [160.5-181.7] | 0.533 | 0.6% |
|  | **T_2_ρ map [ms]** | 80.0 [74.1-88.0] | 78.6 [73.8-86.7] | 0.562 | 1.7% |
|  | **T_1_ map [ms]** | 1345.3 [1303.6-1395.7] | 1347.4 [1307.3-1399.8] | 0.482 | -0.2% |
|  | **T_2_ map [ms]** | 102.0 [93.4-115.5] | 100.0 [92.3-113.0] | 0.570 | 2.0% |
|  | **NODDI NDI** | 0.412 [0.392-0.429] | 0.411 [0.394-0.433] | 0.481 | 0.1% |
|  | **NODDI ISO** | 0.002 [0.000-0.009] | 0.002 [0.000-0.010] | 0.507 | -1.2% |
|  | **NODDI ODI** | 0.573 [0.524-0.616] | 0.578 [0.530-0.622] | 0.454 | -1.0% |
| Paracentral Lobular and Mid Cingulate Cortex | **T_1_ρ map [ms]** | 171.3 [160.8-187.8] | 170.6 [160.1-185.1] | 0.530 | 0.4% |
|  | **T_2_ρ map [ms]** | 82.7 [75.6-96.7] | 83.7 [75.7-96.9] | 0.488 | -1.2% |
|  | **T_1_ map [ms]** | 1359.9 [1290.6-1428.0] | 1362.9 [1290.0-1430.7] | 0.488 | -0.2% |
|  | **T_2_ map [ms]** | 102.6 [93.1-119.2] | 102.2 [92.2-118.1] | 0.509 | 0.5% |
|  | **NODDI NDI** | 0.422 [0.388-0.454] | 0.422 [0.389-0.457] | 0.506 | 0.1% |
|  | **NODDI ISO** | 0.010 [0.001-0.031] | 0.010 [0.001-0.031] | 0.496 | -5.4% |
|  | **NODDI ODI** | 0.573 [0.520-0.626] | 0.574 [0.523-0.631] | 0.485 | -0.3% |
| Early Auditory Cortex | **T_1_ρ map [ms]** | 165.6 [156.9-178.0] | 165.1 [156.4-177.2] | 0.505 | 0.3% |
|  | **T_2_ρ map [ms]** | 79.1 [73.4-87.9] | 77.9 [72.7-85.5] | 0.557 | 1.5% |
|  | **T_1_ map [ms]** | 1274.6 [1215.9-1324.3] | 1278.6 [1228.1-1327.0] | 0.464 | -0.3% |
|  | **T_2_ map [ms]** | 94.6 [86.8-107.7] | 93.2 [86.1-106.1] | 0.537 | 1.4% |
|  | **NODDI NDI** | 0.440 [0.415-0.474] | 0.440 [0.417-0.476] | 0.497 | 0.1% |
|  | **NODDI ISO** | 0.010 [0.002-0.037] | 0.008 [0.001-0.038] | 0.521 | 15.1% |
|  | **NODDI ODI** | 0.597 [0.543-0.651] | 0.605 [0.558-0.658] | 0.440 | -1.2% |
| Auditory Association Cortex | **T_1_ρ map [ms]** | 167.7 [157.4-187.4] | 167.9 [157.7-187.4] | 0.503 | -0.1% |
|  | **T_2_ρ map [ms]** | 78.3 [72.3-89.3] | 78.0 [72.1-89.0] | 0.517 | 0.4% |
|  | **T_1_ map [ms]** | 1375.1 [1312.5-1435.4] | 1376.4 [1316.3-1436.2] | 0.490 | -0.1% |
|  | **T_2_ map [ms]** | 98.7 [90.4-112.6] | 98.1 [89.3-111.2] | 0.530 | 0.6% |
|  | **NODDI NDI** | 0.412 [0.390-0.428] | 0.412 [0.389-0.432] | 0.488 | 0.0% |
|  | **NODDI ISO** | 0.001 [0.000-0.007] | 0.001 [0.000-0.009] | 0.489 | -5.3% |
|  | **NODDI ODI** | 0.583 [0.526-0.627] | 0.582 [0.527-0.627] | 0.497 | 0.2% |
| Insular and Frontal Opercular Cortex | **T_1_ρ map [ms]** | 173.8 [163.8-186.5] | 173.7 [164.5-185.5] | 0.504 | 0.1% |
|  | **T_2_ρ map [ms]** | 80.7 [75.0-88.1] | 80.2 [74.9-86.7] | 0.532 | 0.5% |
|  | **T_1_ map [ms]** | 1402.9 [1341.8-1458.8] | 1404.5 [1345.2-1458.3] | 0.489 | -0.1% |
|  | **T_2_ map [ms]** | 101.0 [93.6-113.0] | 100.0 [93.0-110.9] | 0.543 | 1.0% |
|  | **NODDI NDI** | 0.409 [0.384-0.432] | 0.409 [0.386-0.435] | 0.489 | 0.1% |
|  | **NODDI ISO** | 0.004 [0.000-0.030] | 0.005 [0.001-0.037] | 0.481 | -10.8% |
|  | **NODDI ODI** | 0.540 [0.435-0.606] | 0.541 [0.443-0.607] | 0.490 | -0.2% |
| Medial Temporal Cortex | **T_1_ρ map [ms]** | 167.7 [158.8-178.5] | 166.8 [158.5-177.8] | 0.534 | 0.5% |
|  | **T_2_ρ map [ms]** | 80.3 [74.0-90.5] | 79.3 [73.6-89.3] | 0.537 | 1.3% |
|  | **T_1_ map [ms]** | 1353.8 [1234.2-1428.7] | 1354.9 [1240.8-1427.0] | 0.502 | -0.1% |
|  | **T_2_ map [ms]** | 94.5 [86.7-110.2] | 93.5 [85.9-106.1] | 0.554 | 1.1% |
|  | **NODDI NDI** | 0.445 [0.415-0.520] | 0.445 [0.418-0.520] | 0.490 | 0.0% |
|  | **NODDI ISO** | 0.033 [0.000-0.138] | 0.032 [0.001-0.128] | 0.504 | 1.5% |
|  | **NODDI ODI** | 0.592 [0.471-0.649] | 0.592 [0.478-0.648] | 0.498 | 0.0% |
| Lateral Temporal Cortex | **T_1_ρ map [ms]** | 164.0 [157.2-175.0] | 164.1 [157.2-176.6] | 0.494 | 0.0% |
|  | **T_2_ρ map [ms]** | 75.4 [72.0-81.1] | 75.2 [71.3-80.7] | 0.536 | 0.3% |
|  | **T_1_ map [ms]** | 1414.6 [1349.9-1464.4] | 1415.8 [1351.1-1464.0] | 0.498 | -0.1% |
|  | **T_2_ map [ms]** | 95.0 [89.5-104.8] | 94.7 [88.1-102.7] | 0.540 | 0.4% |
|  | **NODDI NDI** | 0.415 [0.391-0.475] | 0.421 [0.391-0.486] | 0.464 | -1.3% |
|  | **NODDI ISO** | 0.006 [0.000-0.033] | 0.008 [0.000-0.038] | 0.468 | -20.2% |
|  | **NODDI ODI** | 0.600 [0.546-0.650] | 0.603 [0.549-0.648] | 0.477 | -0.5% |
| Temporal-Parietal-Occipital Junction | **T_1_ρ map [ms]** | 161.0 [154.4-172.2] | 161.5 [154.5-170.0] | 0.491 | -0.3% |
|  | **T_2_ρ map [ms]** | 74.7 [70.8-81.7] | 74.6 [70.4-79.6] | 0.529 | 0.1% |
|  | **T_1_ map [ms]** | 1350.2 [1306.0-1397.5] | 1354.8 [1306.7-1401.8] | 0.470 | -0.3% |
|  | **T_2_ map [ms]** | 94.3 [88.2-103.7] | 94.4 [88.2-102.7] | 0.509 | -0.1% |
|  | **NODDI NDI** | 0.408 [0.389-0.423] | 0.407 [0.389-0.425] | 0.509 | 0.2% |
|  | **NODDI ISO** | 0.000 [0.000-0.003] | 0.001 [0.000-0.003] | 0.457 | -19.9% |
|  | **NODDI ODI** | 0.588 [0.542-0.632] | 0.592 [0.545-0.636] | 0.466 | -0.6% |
| Superior Parietal and IPS | **T_1_ρ map [ms]** | 174.5 [160.7-189.3] | 174.3 [161.2-192.1] | 0.487 | 0.1% |
|  | **T_2_ρ map [ms]** | 85.1 [75.6-96.0] | 85.0 [76.0-97.5] | 0.488 | 0.1% |
|  | **T_1_ map [ms]** | 1348.5 [1291.7-1409.6] | 1350.6 [1300.7-1408.1] | 0.480 | -0.2% |
|  | **T_2_ map [ms]** | 101.3 [90.7-117.2] | 101.5 [90.7-115.4] | 0.506 | -0.2% |
|  | **NODDI NDI** | 0.395 [0.371-0.420] | 0.394 [0.370-0.419] | 0.517 | 0.3% |
|  | **NODDI ISO** | 0.003 [0.000-0.012] | 0.003 [0.000-0.013] | 0.496 | 5.5% |
|  | **NODDI ODI** | 0.571 [0.520-0.619] | 0.571 [0.512-0.621] | 0.501 | 0.0% |
| Inferior Parietal Cortex | **T_1_ρ map [ms]** | 166.6 [157.9-177.1] | 166.1 [158.4-175.4] | 0.516 | 0.3% |
|  | **T_2_ρ map [ms]** | 78.7 [73.5-85.7] | 78.0 [73.0-85.3] | 0.544 | 0.9% |
|  | **T_1_ map [ms]** | 1368.5 [1317.6-1419.6] | 1369.7 [1323.6-1420.5] | 0.490 | -0.1% |
|  | **T_2_ map [ms]** | 96.8 [90.1-104.9] | 96.2 [90.2-102.9] | 0.536 | 0.7% |
|  | **NODDI NDI** | 0.402 [0.383-0.423] | 0.401 [0.384-0.423] | 0.513 | 0.3% |
|  | **NODDI ISO** | 0.001 [0.000-0.005] | 0.001 [0.000-0.005] | 0.528 | 15.7% |
|  | **NODDI ODI** | 0.574 [0.538-0.615] | 0.574 [0.537-0.613] | 0.505 | 0.1% |
| Posterior Cingulate Cortex | **T_1_ρ map [ms]** | 162.7 [154.3-172.9] | 162.4 [154.2-172.5] | 0.504 | 0.2% |
|  | **T_2_ρ map [ms]** | 76.2 [71.8-82.5] | 75.9 [71.2-82.2] | 0.524 | 0.4% |
|  | **T_1_ map [ms]** | 1346.3 [1283.1-1395.3] | 1345.2 [1288.7-1393.5] | 0.504 | 0.1% |
|  | **T_2_ map [ms]** | 93.3 [86.2-103.3] | 92.8 [85.1-101.9] | 0.534 | 0.5% |
|  | **NODDI NDI** | 0.419 [0.396-0.445] | 0.418 [0.396-0.449] | 0.506 | 0.0% |
|  | **NODDI ISO** | 0.003 [0.000-0.017] | 0.003 [0.000-0.019] | 0.488 | -6.3% |
|  | **NODDI ODI** | 0.576 [0.527-0.620] | 0.577 [0.526-0.623] | 0.492 | -0.3% |
| Anterior Cingulate Cortex | **T_1_ρ map [ms]** | 171.2 [161.3-182.8] | 170.2 [161.3-181.1] | 0.534 | 0.6% |
|  | **T_2_ρ map [ms]** | 79.1 [73.7-86.0] | 78.6 [73.5-85.2] | 0.536 | 0.6% |
|  | **T_1_ map [ms]** | 1441.6 [1389.8-1489.9] | 1439.2 [1393.2-1488.2] | 0.503 | 0.2% |
|  | **T_2_ map [ms]** | 102.0 [94.4-115.1] | 101.2 [93.6-112.6] | 0.538 | 0.9% |
|  | **NODDI NDI** | 0.416 [0.395-0.475] | 0.415 [0.393-0.492] | 0.508 | 0.3% |
|  | **NODDI ISO** | 0.004 [0.000-0.043] | 0.003 [0.000-0.056] | 0.508 | 9.3% |
|  | **NODDI ODI** | 0.591 [0.533-0.678] | 0.590 [0.534-0.689] | 0.493 | 0.2% |
| Orbital and Polar Frontal Cortex | **T_1_ρ map [ms]** | 166.2 [156.4-182.5] | 165.3 [156.7-180.5] | 0.529 | 0.5% |
|  | **T_2_ρ map [ms]** | 77.4 [71.9-87.7] | 76.9 [71.6-86.8] | 0.521 | 0.6% |
|  | **T_1_ map [ms]** | 1424.0 [1357.1-1482.6] | 1422.4 [1360.2-1480.9] | 0.500 | 0.1% |
|  | **T_2_ map [ms]** | 105.3 [96.5-119.1] | 104.4 [95.5-117.9] | 0.532 | 0.9% |
|  | **NODDI NDI** | 0.418 [0.377-0.587] | 0.417 [0.379-0.615] | 0.495 | 0.2% |
|  | **NODDI ISO** | 0.011 [0.001-0.103] | 0.011 [0.001-0.115] | 0.498 | -5.7% |
|  | **NODDI ODI** | 0.603 [0.548-0.678] | 0.606 [0.554-0.686] | 0.468 | -0.5% |
| Inferior Frontal Cortex | **T_1_ρ map [ms]** | 167.3 [155.3-180.6] | 167.1 [155.2-179.8] | 0.506 | 0.1% |
|  | **T_2_ρ map [ms]** | 79.1 [72.1-87.8] | 78.9 [72.5-87.6] | 0.510 | 0.3% |
|  | **T_1_ map [ms]** | 1374.7 [1323.0-1434.4] | 1379.0 [1332.8-1440.7] | 0.464 | -0.3% |
|  | **T_2_ map [ms]** | 100.0 [91.2-110.0] | 100.1 [91.5-109.5] | 0.502 | -0.1% |
|  | **NODDI NDI** | 0.412 [0.392-0.438] | 0.411 [0.390-0.438] | 0.514 | 0.3% |
|  | **NODDI ISO** | 0.003 [0.000-0.014] | 0.003 [0.000-0.016] | 0.509 | 8.4% |
|  | **NODDI ODI** | 0.581 [0.535-0.624] | 0.581 [0.534-0.622] | 0.497 | 0.0% |
| Dorsolateral Prefrontal Cortex | **T_1_ρ map [ms]** | 167.4 [153.9-181.0] | 165.7 [152.6-176.7] | 0.560 | 1.1% |
|  | **T_2_ρ map [ms]** | 82.1 [75.6-92.4] | 81.6 [75.8-91.3] | 0.518 | 0.6% |
|  | **T_1_ map [ms]** | 1427.6 [1368.8-1486.3] | 1430.6 [1380.1-1487.4] | 0.481 | -0.2% |
|  | **T_2_ map [ms]** | 106.1 [97.2-118.3] | 105.6 [97.7-117.8] | 0.516 | 0.4% |
|  | **NODDI NDI** | 0.411 [0.388-0.436] | 0.408 [0.385-0.436] | 0.535 | 0.7% |
|  | **NODDI ISO** | 0.006 [0.001-0.025] | 0.005 [0.001-0.024] | 0.531 | 18.3% |
|  | **NODDI ODI** | 0.568 [0.519-0.605] | 0.568 [0.525-0.606] | 0.482 | -0.1% |
| Thalamus | **T_1_ρ map [ms]** | 159.6 [146.1-206.8] | 160.0 [145.8-199.3] | 0.501 | -0.3% |
|  | **T_2_ρ map [ms]** | 82.1 [73.9-110.9] | 81.6 [74.2-105.4] | 0.514 | 0.6% |
|  | **T_1_ map [ms]** | 1030.1 [953.3-1230.7] | 1024.1 [956.0-1218.1] | 0.508 | 0.6% |
|  | **T_2_ map [ms]** | 98.6 [82.5-171.8] | 97.6 [81.4-162.4] | 0.510 | 1.0% |
|  | **NODDI NDI** | 0.630 [0.574-0.700] | 0.631 [0.569-0.689] | 0.518 | -0.1% |
|  | **NODDI ISO** | 0.105 [0.064-0.231] | 0.106 [0.070-0.220] | 0.487 | -1.2% |
|  | **NODDI ODI** | 0.332 [0.280-0.395] | 0.334 [0.279-0.399] | 0.493 | -0.6% |
| Hypothalamus | **T_1_ρ map [ms]** | 211.1 [160.8-265.0] | 208.8 [159.1-258.2] | 0.527 | 1.1% |
|  | **T_2_ρ map [ms]** | 108.8 [75.4-142.6] | 108.0 [75.6-140.1] | 0.522 | 0.7% |
|  | **T_1_ map [ms]** | 1312.0 [1132.7-1440.9] | 1307.8 [1128.3-1433.2] | 0.517 | 0.3% |
|  | **T_2_ map [ms]** | 146.7 [93.5-232.3] | 141.1 [90.6-221.4] | 0.533 | 4.0% |
|  | **NODDI NDI** | 0.520 [0.431-0.670] | 0.526 [0.442-0.667] | 0.485 | -1.1% |
|  | **NODDI ISO** | 0.192 [0.052-0.434] | 0.180 [0.049-0.420] | 0.502 | 6.7% |
|  | **NODDI ODI** | 0.427 [0.361-0.519] | 0.455 [0.369-0.542] | 0.404 | -6.2% |
| Striatum | **T_1_ρ map [ms]** | 158.9 [144.4-192.7] | 159.5 [145.0-186.9] | 0.492 | -0.4% |
|  | **T_2_ρ map [ms]** | 68.0 [59.6-92.4] | 69.1 [60.4-86.4] | 0.493 | -1.5% |
|  | **T_1_ map [ms]** | 1197.2 [1078.2-1299.7] | 1190.0 [1078.9-1283.2] | 0.514 | 0.6% |
|  | **T_2_ map [ms]** | 72.5 [63.8-104.8] | 73.6 [64.3-96.7] | 0.499 | -1.4% |
|  | **NODDI NDI** | 0.536 [0.491-0.625] | 0.530 [0.487-0.610] | 0.540 | 1.2% |
|  | **NODDI ISO** | 0.023 [0.004-0.119] | 0.020 [0.003-0.099] | 0.525 | 14.6% |
|  | **NODDI ODI** | 0.602 [0.538-0.661] | 0.599 [0.539-0.662] | 0.516 | 0.5% |
| Pallidum | **T_1_ρ map [ms]** | 134.9 [131.4-139.2] | 135.1 [131.6-139.6] | 0.473 | -0.2% |
|  | **T_2_ρ map [ms]** | 60.1 [56.5-64.8] | 60.6 [57.2-64.8] | 0.469 | -0.9% |
|  | **T_1_ map [ms]** | 899.2 [867.3-932.2] | 900.1 [869.0-937.5] | 0.462 | -0.1% |
|  | **T_2_ map [ms]** | 60.0 [54.0-66.1] | 60.5 [54.5-66.9] | 0.438 | -0.9% |
|  | **NODDI NDI** | 0.952 [0.862-0.990] | 0.936 [0.838-0.987] | 0.565 | 1.6% |
|  | **NODDI ISO** | 0.150 [0.080-0.203] | 0.164 [0.108-0.206] | 0.404 | -8.5% |
|  | **NODDI ODI** | 0.497 [0.379-0.583] | 0.483 [0.378-0.572] | 0.537 | 3.0% |
| Basal Ganglia-Associated Nuclei | **T_1_ρ map [ms]** | 138.9 [129.2-149.8] | 139.0 [129.9-149.9] | 0.496 | -0.1% |
|  | **T_2_ρ map [ms]** | 67.7 [63.6-72.7] | 67.0 [63.7-72.9] | 0.499 | 1.0% |
|  | **T_1_ map [ms]** | 890.4 [832.6-952.2] | 900.0 [832.6-960.8] | 0.478 | -1.1% |
|  | **T_2_ map [ms]** | 64.5 [57.4-71.1] | 64.9 [58.7-70.2] | 0.497 | -0.6% |
|  | **NODDI NDI** | 0.933 [0.844-0.978] | 0.917 [0.799-0.976] | 0.558 | 1.7% |
|  | **NODDI ISO** | 0.153 [0.091-0.222] | 0.171 [0.100-0.255] | 0.409 | -10.3% |
|  | **NODDI ODI** | 0.372 [0.287-0.443] | 0.359 [0.291-0.432] | 0.535 | 3.6% |
| Monoaminergic and Arousal Nuclei | **T_1_ρ map [ms]** | 168.2 [155.0-194.8] | 169.0 [155.6-193.8] | 0.504 | -0.5% |
|  | **T_2_ρ map [ms]** | 99.4 [89.5-118.9] | 99.5 [88.2-114.1] | 0.533 | -0.1% |
|  | **T_1_ map [ms]** | 1019.3 [977.9-1094.1] | 1020.4 [979.4-1085.8] | 0.488 | -0.1% |
|  | **T_2_ map [ms]** | 85.4 [75.6-140.6] | 84.1 [74.9-141.4] | 0.526 | 1.6% |
|  | **NODDI NDI** | 0.605 [0.517-0.729] | 0.621 [0.516-0.728] | 0.479 | -2.5% |
|  | **NODDI ISO** | 0.160 [0.069-0.268] | 0.167 [0.058-0.330] | 0.456 | -4.3% |
|  | **NODDI ODI** | 0.213 [0.145-0.374] | 0.222 [0.138-0.371] | 0.500 | -4.0% |
| Reticular and Sensorimotor Integration Nuclei | **T_1_ρ map [ms]** | 149.5 [141.7-189.4] | 149.6 [141.5-188.0] | 0.510 | -0.1% |
|  | **T_2_ρ map [ms]** | 79.8 [71.2-100.6] | 79.2 [71.2-101.2] | 0.511 | 0.8% |
|  | **T_1_ map [ms]** | 966.0 [877.5-1313.6] | 967.1 [880.6-1314.5] | 0.490 | -0.1% |
|  | **T_2_ map [ms]** | 80.0 [68.5-106.8] | 79.2 [67.7-104.1] | 0.511 | 1.0% |
|  | **NODDI NDI** | 0.740 [0.498-0.907] | 0.737 [0.498-0.899] | 0.518 | 0.4% |
|  | **NODDI ISO** | 0.134 [0.026-0.203] | 0.141 [0.028-0.214] | 0.463 | -4.7% |
|  | **NODDI ODI** | 0.334 [0.194-0.410] | 0.340 [0.194-0.417] | 0.503 | -1.7% |
| Amygdala | **T_1_ρ map [ms]** | 177.8 [169.9-192.4] | 178.0 [169.5-192.7] | 0.497 | -0.1% |
|  | **T_2_ρ map [ms]** | 83.6 [79.1-92.6] | 83.6 [78.6-91.2] | 0.507 | -0.1% |
|  | **T_1_ map [ms]** | 1345.0 [1283.3-1430.9] | 1343.2 [1286.1-1409.9] | 0.517 | 0.1% |
|  | **T_2_ map [ms]** | 97.8 [89.6-138.6] | 96.9 [88.7-130.9] | 0.525 | 1.0% |
|  | **NODDI NDI** | 0.432 [0.406-0.470] | 0.429 [0.400-0.463] | 0.548 | 0.8% |
|  | **NODDI ISO** | 0.034 [0.009-0.106] | 0.032 [0.009-0.089] | 0.529 | 5.9% |
|  | **NODDI ODI** | 0.514 [0.431-0.589] | 0.512 [0.428-0.591] | 0.513 | 0.4% |
| Hippocampus | **T_1_ρ map [ms]** | 167.4 [157.4-184.1] | 166.6 [156.7-182.5] | 0.522 | 0.5% |
|  | **T_2_ρ map [ms]** | 80.3 [75.0-90.3] | 79.5 [74.5-89.0] | 0.543 | 1.0% |
|  | **T_1_ map [ms]** | 1323.5 [1139.3-1417.0] | 1322.4 [1133.9-1407.4] | 0.515 | 0.1% |
|  | **T_2_ map [ms]** | 101.5 [86.1-136.2] | 100.0 [84.8-127.1] | 0.535 | 1.6% |
|  | **NODDI NDI** | 0.449 [0.398-0.545] | 0.446 [0.398-0.542] | 0.509 | 0.8% |
|  | **NODDI ISO** | 0.057 [0.016-0.178] | 0.054 [0.015-0.159] | 0.518 | 5.1% |
|  | **NODDI ODI** | 0.546 [0.393-0.637] | 0.538 [0.390-0.641] | 0.514 | 1.5% |
| Cerebellar Cortex | **T_1_ρ map [ms]** | 180.5 [164.6-213.3] | 179.0 [164.5-212.8] | 0.523 | 0.8% |
|  | **T_2_ρ map [ms]** | 87.1 [75.9-110.9] | 85.8 [75.0-109.7] | 0.530 | 1.5% |
|  | **T_1_ map [ms]** | 1361.1 [1290.6-1482.7] | 1353.0 [1285.0-1471.6] | 0.533 | 0.6% |
|  | **T_2_ map [ms]** | 118.2 [97.1-184.5] | 115.9 [94.8-180.9] | 0.528 | 2.0% |
|  | **NODDI NDI** | 0.559 [0.495-0.673] | 0.553 [0.489-0.661] | 0.526 | 1.1% |
|  | **NODDI ISO** | 0.087 [0.032-0.271] | 0.077 [0.027-0.254] | 0.536 | 13.0% |
|  | **NODDI ODI** | 0.498 [0.444-0.558] | 0.496 [0.437-0.553] | 0.521 | 0.3% |
| Cerebellar Deep Nuclei | **T_1_ρ map [ms]** | 146.7 [137.4-155.7] | 146.8 [137.6-156.3] | 0.480 | -0.1% |
|  | **T_2_ρ map [ms]** | 74.5 [69.6-81.6] | 75.4 [69.9-82.2] | 0.484 | -1.2% |
|  | **T_1_ map [ms]** | 919.4 [883.0-954.5] | 922.0 [888.1-965.0] | 0.453 | -0.3% |
|  | **T_2_ map [ms]** | 83.6 [75.6-91.9] | 84.0 [76.0-92.2] | 0.494 | -0.4% |
|  | **NODDI NDI** | 0.724 [0.635-0.822] | 0.710 [0.609-0.797] | 0.552 | 2.0% |
|  | **NODDI ISO** | 0.109 [0.079-0.138] | 0.107 [0.072-0.132] | 0.537 | 1.7% |
|  | **NODDI ODI** | 0.364 [0.310-0.410] | 0.371 [0.309-0.408] | 0.476 | -1.8% |
| White Matter – Commissural Fibres | **T_1_ρ map [ms]** | 157.4 [141.4-173.1] | 156.8 [141.0-173.0] | 0.509 | 0.4% |
|  | **T_2_ρ map [ms]** | 83.2 [72.3-93.4] | 83.2 [72.7-93.2] | 0.499 | 0.0% |
|  | **T_1_ map [ms]** | 944.0 [862.6-1065.4] | 951.4 [863.7-1077.0] | 0.496 | -0.8% |
|  | **T_2_ map [ms]** | 97.4 [80.1-118.9] | 98.3 [81.3-116.6] | 0.501 | -0.8% |
|  | **NODDI NDI** | 0.678 [0.610-0.795] | 0.676 [0.607-0.787] | 0.503 | 0.3% |
|  | **NODDI ISO** | 0.154 [0.120-0.191] | 0.153 [0.118-0.185] | 0.516 | 1.0% |
|  | **NODDI ODI** | 0.256 [0.138-0.339] | 0.252 [0.135-0.378] | 0.497 | 1.9% |
| White Matter – Association Fibres | **T_1_ρ map [ms]** | 140.3 [135.5-145.8] | 140.0 [134.5-145.2] | 0.541 | 0.3% |
|  | **T_2_ρ map [ms]** | 72.7 [70.3-76.0] | 72.5 [69.8-75.3] | 0.537 | 0.3% |
|  | **T_1_ map [ms]** | 880.1 [841.5-937.4] | 878.3 [841.0-927.5] | 0.518 | 0.2% |
|  | **T_2_ map [ms]** | 85.3 [80.2-91.0] | 84.9 [80.2-89.8] | 0.537 | 0.5% |
|  | **NODDI NDI** | 0.624 [0.556-0.694] | 0.634 [0.564-0.693] | 0.472 | -1.5% |
|  | **NODDI ISO** | 0.094 [0.064-0.121] | 0.094 [0.067-0.115] | 0.505 | -0.3% |
|  | **NODDI ODI** | 0.248 [0.202-0.282] | 0.248 [0.201-0.281] | 0.511 | 0.2% |
| White Matter – Projection Fibres | **T_1_ρ map [ms]** | 143.0 [137.2-149.7] | 142.9 [136.6-148.7] | 0.524 | 0.1% |
|  | **T_2_ρ map [ms]** | 75.2 [70.1-80.8] | 75.0 [70.0-80.7] | 0.517 | 0.3% |
|  | **T_1_ map [ms]** | 892.4 [846.8-935.0] | 892.5 [847.8-929.9] | 0.507 | 0.0% |
|  | **T_2_ map [ms]** | 87.7 [79.1-95.6] | 87.4 [78.3-95.0] | 0.523 | 0.4% |
|  | **NODDI NDI** | 0.648 [0.587-0.760] | 0.648 [0.592-0.756] | 0.493 | -0.1% |
|  | **NODDI ISO** | 0.111 [0.082-0.143] | 0.109 [0.085-0.144] | 0.502 | 1.4% |
|  | **NODDI ODI** | 0.237 [0.195-0.296] | 0.240 [0.193-0.295] | 0.506 | -1.3% |
| White Matter – Limbic Fibres | **T_1_ρ map [ms]** | 151.3 [138.0-197.4] | 150.2 [137.6-198.5] | 0.518 | 0.8% |
|  | **T_2_ρ map [ms]** | 75.2 [69.4-106.3] | 74.4 [69.2-107.0] | 0.528 | 1.2% |
|  | **T_1_ map [ms]** | 940.6 [867.8-1226.8] | 933.5 [865.8-1238.1] | 0.515 | 0.8% |
|  | **T_2_ map [ms]** | 84.6 [77.2-172.2] | 83.2 [76.5-172.0] | 0.529 | 1.8% |
|  | **NODDI NDI** | 0.586 [0.511-0.675] | 0.591 [0.519-0.671] | 0.485 | -0.8% |
|  | **NODDI ISO** | 0.098 [0.052-0.355] | 0.097 [0.057-0.345] | 0.508 | 1.0% |
|  | **NODDI ODI** | 0.243 [0.194-0.301] | 0.241 [0.190-0.300] | 0.507 | 1.2% |

**Supplementary Table 4: Pooled summary of top parcels contributing to the Brain–Cognition axis** (τ = 0.50), i.e. the smallest set of parcels whose absolute discriminant weights covered ≥50% of the within-metric L_1_ norm. Pooled by overarching anatomical category (see Supplementary Table 1). Signed weight sum corresponds to signed sum of parcel weights in the category (direction relative to the axis). L1 share is the percentage of the within-metric magnitude (L1) contributed by that pooled category.

| Metric | Region | Parcels (n) | Signed weight sum | L1 share (%) |
| --- | --- | --- | --- | --- |
| Isotropic compartment fraction (ISO) ~55% of the MRI block’s absolute discriminant weight (‖*w*‖_1_) | Superior Parietal and IPS | 18 | 10.301 | 8.0 |
|  | Dorsolateral Prefrontal Cortex | 15 | 7.996 | 6.2 |
|  | Inferior Parietal Cortex | 9 | 3.578 | 3.4 |
|  | Orbital and Polar Frontal Cortex | 9 | 4.059 | 3.2 |
|  | MT+ Complex and Neighboring Visual Areas | 8 | 1.168 | 2.9 |
|  | Paracentral Lobular and Mid Cingulate Cortex | 8 | 3.541 | 2.8 |
|  | Posterior Cingulate | 8 | 1.853 | 2.7 |
|  | Lateral Temporal Cortex | 6 | 3.133 | 2.4 |
|  | Somatosensory and Motor Cortex | 5 | 2.581 | 2.0 |
|  | Cerebellum | 4 | 2.074 | 1.6 |
|  | Premotor Cortex | 4 | 1.955 | 1.5 |
|  | Dorsal Stream Visual Cortex | 4 | 1.213 | 1.5 |
|  | Auditory Association Cortex | 4 | 0.910 | 1.4 |
|  | Ventral Stream Visual Cortex | 4 | -1.540 | 1.2 |
|  | Posterior Operculum | 3 | -1.373 | 1.1 |
|  | Early Visual Cortex | 3 | 1.364 | 1.1 |
|  | Cerebellar Deep Nuclei | 3 | -1.292 | 1.0 |
|  | Anterior Cingulate | 2 | 1.218 | 1.0 |
|  | White Matter Association Fibres | 3 | -0.372 | 1.0 |
|  | Inferior Frontal Cortex | 2 | 0.982 | 0.8 |
|  | Insular Cortex | 2 | 0.058 | 0.7 |
|  | Primary Visual Cortex | 2 | 0.808 | 0.6 |
|  | Temporal-Parietal-Occipital Junction | 2 | -0.730 | 0.6 |
|  | White Matter Projection Fibres | 1 | 0.449 | 0.4 |
|  | Striatum | 1 | 0.442 | 0.3 |
|  | White Matter Commissural Fibres | 1 | 0.422 | 0.3 |
|  | Thalamus | 1 | 0.360 | 0.3 |
|  | Hippocampus | 1 | -0.344 | 0.3 |
| Neurite density index (NDI) ~42% of the MRI block’s absolute discriminant weight (‖*w*‖_1_) | Orbital and Polar Frontal Cortex | 11 | -5.817 | 5.4 |
|  | Posterior Cingulate | 14 | 5.524 | 5.2 |
|  | Dorsolateral Prefrontal Cortex | 11 | -4.559 | 4.3 |
|  | Cerebellum | 10 | 2.154 | 3.5 |
|  | Insular Cortex | 9 | 2.048 | 3.3 |
|  | Inferior Frontal Cortex | 6 | -3.176 | 3.0 |
|  | MT+ Complex and Neighboring Visual Areas | 5 | -2.606 | 2.4 |
|  | Ventral Stream Visual Cortex | 7 | 1.867 | 2.3 |
|  | Temporal-Parietal-Occipital Junction | 5 | 1.875 | 1.7 |
|  | Anterior Cingulate | 5 | -1.860 | 1.7 |
|  | Posterior Operculum | 5 | 1.045 | 1.7 |
|  | Brainstem Reticular and Sensorimotor Integration Nuclei | 4 | 1.697 | 1.6 |
|  | Auditory Association Cortex | 4 | -0.962 | 1.5 |
|  | Hippocampus | 4 | 1.520 | 1.4 |
|  | Early Visual Cortex | 3 | -1.339 | 1.2 |
|  | Dorsal Stream Visual Cortex | 3 | -1.334 | 1.2 |
|  | Brainstem Monoaminergic and Arousal Nuclei | 3 | 1.156 | 1.1 |
|  | Medial Temporal | 3 | 1.149 | 1.1 |
|  | Lateral Temporal Cortex | 2 | -1.041 | 1.0 |
|  | Amygdala | 2 | 0.774 | 0.7 |
|  | Paracentral Lobular and Mid Cingulate Cortex | 2 | 0.160 | 0.7 |
|  | White Matter Limbic Fibres | 2 | -0.745 | 0.7 |
|  | Primary Visual Cortex | 2 | -0.709 | 0.7 |
|  | Striatum | 2 | -0.669 | 0.6 |
|  | White Matter Projection Fibres | 2 | -0.665 | 0.6 |
|  | Inferior Parietal Cortex | 1 | -0.616 | 0.6 |
|  | Superior Parietal and IPS | 1 | 0.353 | 0.3 |
|  | Premotor Cortex | 1 | -0.316 | 0.3 |
|  | White Matter Association Fibres | 1 | -0.313 | 0.3 |
| T_2_ map ~3% of the MRI block’s absolute discriminant weight (‖*w*‖_1_) | Dorsolateral Prefrontal Cortex | 18 | 1.075 | 6.4 |
|  | Anterior Cingulate | 9 | 0.550 | 3.3 |
|  | Inferior Parietal Cortex | 9 | 0.547 | 3.3 |
|  | Premotor Cortex | 8 | 0.489 | 2.9 |
|  | Somatosensory and Motor Cortex | 7 | 0.423 | 2.5 |
|  | Auditory Association Cortex | 7 | 0.423 | 2.5 |
|  | White Matter Association Fibres | 7 | 0.416 | 2.5 |
|  | Insular Cortex | 6 | 0.392 | 2.4 |
|  | Inferior Frontal Cortex | 6 | 0.380 | 2.3 |
|  | Superior Parietal and IPS | 6 | 0.310 | 1.9 |
|  | Lateral Temporal Cortex | 6 | 0.304 | 1.8 |
|  | Medial Temporal | 5 | 0.295 | 1.8 |
|  | White Matter Limbic Fibres | 4 | 0.259 | 1.6 |
|  | Brainstem Reticular and Sensorimotor Integration Nuclei | 4 | 0.259 | 1.6 |
|  | Striatum | 4 | 0.247 | 1.5 |
|  | Thalamus | 4 | 0.229 | 1.4 |
|  | Early Auditory Cortex | 4 | 0.220 | 1.3 |
|  | Hippocampus | 3 | 0.186 | 1.1 |
|  | White Matter Projection Fibres | 3 | 0.184 | 1.1 |
|  | Orbital and Polar Frontal Cortex | 3 | 0.170 | 1.0 |
|  | Posterior Cingulate | 3 | 0.160 | 1.0 |
|  | Posterior Operculum | 3 | 0.159 | 1.0 |
|  | Paracentral Lobular and Mid Cingulate Cortex | 2 | 0.116 | 0.7 |
|  | Ventral Stream Visual Cortex | 2 | 0.111 | 0.7 |
|  | MT+ Complex and Neighboring Visual Areas | 2 | 0.108 | 0.6 |
|  | Temporal-Parietal-Occipital Junction | 2 | 0.101 | 0.6 |
|  | Amygdala | 1 | 0.071 | 0.4 |
|  | White Matter Commissural Fibres | 1 | 0.062 | 0.4 |
|  | Hypothalamus | 1 | 0.058 | 0.3 |
|  | Brainstem Monoaminergic and Arousal Nuclei | 1 | 0.055 | 0.3 |

**Supplementary Table 5: Association of the Brain–Cognition axis with Newcastle score and its components**. Regression coefficients β and partial R^2^ values (age- and sex-adjusted) quantify the unique variance in each outcome explained by the axis. p-values are from 10,000 covariate-preserving permutations; q is Benjamini–Hochberg FDR across the four outcomes. The GAM effective degrees of freedom (EDF) and smooth p-values assess non-linearity; EDF ≈ 1 with small p indicates that a linear term suffices. All domains show significant positive associations, strongest for the full Newcastle score. Abbreviations: BH – Benjamini-Hochberg False Discovery Rate correction; EDF – Effective Degrees of Freedom; GAM – Generalised Additive Model

| Domain | *β* | partial R^2^ | p value | q (BH) | EDF (GAM) | p (smooth) |
| --- | --- | --- | --- | --- | --- | --- |
| Full Newcastle score | 0.400 | 0.178 | <0.001 | <0.001 | 1.000 | 0.000 |
| Respiratory component | 0.221 | 0.102 | 0.002 | 0.002 | 1.000 | 0.001 |
| Systemic component | 0.262 | 0.137 | <0.001 | <0.001 | 1.000 | 0.000 |
| Neurological / sensory-autonomic component | 0.237 | 0.134 | <0.001 | 0.001 | 1.000 | 0.000 |

**Supplementary Figures**

**Supplementary Figure 1: Study flow chart (CONSORT-style)** documenting the enrolment process, MRI and neuropsychological assessment (not performed in fixed order), with reasons for exclusion at each step. Participants were classified as “indeterminate” when the documented self-reported symptoms were inconsistent with Newcastle questionnaire responses. In two subjects, only the final sequence of the MRI acquisition was missing/incomplete (T_2_ρ). These participants were retained and their T_2_ρ values imputed as described in Methods. The **complete-case cohort** includes participants with both full MRI and neuropsychology datasets of acceptable quality. Abbreviations: MRI – magnetic resonance imaging.

**Supplementary Figure 2: Parcel-wise loading maps for the five retained** **components of Bayesian probabilistic principal component analysis** (PPCA) with the largest contributions (rows; labelled by metric and component index). Left panels show lateral and medial cortical surfaces; right panels show subcortical axial slices in Montreal Neurological Space [z = 28, 16, 4, -8, -20, -32]. Images follow neurological convention (right = right). Colours depict PPCA loading weights; signs are shown as estimated (no re-orientation), so warm vs cool colours indicate opposite directions within a component but carry no absolute meaning across components. The scale bar reflects the relative magnitude of loadings.

**Supplementary References**

1.	Glasser MF, Coalson TS, Robinson EC, Hacker CD, Harwell J, Yacoub E, et al. A multi-modal parcellation of human cerebral cortex. Nature. 2016;536(7615):171–8.

2.	Behrens TEJ, Johansen-Berg H, Woolrich MW, Smith SM, Wheeler-Kingshott C a. M, Boulby PA, et al. Non-invasive mapping of connections between human thalamus and cortex using diffusion imaging. Nat Neurosci. 2003 Jul;6(7):750–7. doi:10.1038/nn1075 PubMed PMID: 12808459.

3.	Spindler M, Özyurt J, Thiel CM. Automated diffusion-based parcellation of the hypothalamus reveals subunit-specific associations with obesity. Sci Rep. 2020 Dec 17;10(1):22238. doi:10.1038/s41598-020-79289-9

4.	Tziortzi AC, Haber SN, Searle GE, Tsoumpas C, Long CJ, Shotbolt P, et al. Connectivity-Based Functional Analysis of Dopamine Release in the Striatum Using Diffusion-Weighted MRI and Positron Emission Tomography. Cereb Cortex. 2014 May 1;24(5):1165–77. doi:10.1093/cercor/bhs397

5.	Keuken MC, Bazin PL, Crown L, Hootsmans J, Laufer A, Müller-Axt C, et al. Quantifying inter-individual anatomical variability in the subcortex using 7 T structural MRI. NeuroImage. 2014 Jul 1;94:40–6. doi:10.1016/j.neuroimage.2014.03.032 PubMed PMID: 24650599.

6.	Edlow BL, Takahashi E, Wu O, Benner T, Dai G, Bu L, et al. Neuroanatomic connectivity of the human ascending arousal system critical to consciousness and its disorders. J Neuropathol Exp Neurol. 2012 Jun;71(6):531–46. doi:10.1097/NEN.0b013e3182588293 PubMed PMID: 22592840; PubMed Central PMCID: PMC3387430.

7.	Saygin ZM, Kliemann D, Iglesias JE, van der Kouwe AJW, Boyd E, Reuter M, et al. High-resolution magnetic resonance imaging reveals nuclei of the human amygdala: manual segmentation to automatic atlas. NeuroImage. 2017 Jul 15;155:370–82. doi:10.1016/j.neuroimage.2017.04.046 PubMed PMID: 28479476; PubMed Central PMCID: PMC5557007.

8.	Winterburn JL, Pruessner JC, Chavez S, Schira MM, Lobaugh NJ, Voineskos AN, et al. A novel in vivo atlas of human hippocampal subfields using high-resolution 3 T magnetic resonance imaging. NeuroImage. 2013 Jul 1;74:254–65. doi:10.1016/j.neuroimage.2013.02.003 PubMed PMID: 23415948.

9.	Diedrichsen J, Balsters JH, Flavell J, Cussans E, Ramnani N. A probabilistic MR atlas of the human cerebellum. NeuroImage. 2009;46(1):39–46. doi:10.1016/j.neuroimage.2009.01.045

10.	Warrington S, Bryant KL, Khrapitchev AA, Sallet J, Charquero-Ballester M, Douaud G, et al. XTRACT - Standardised protocols for automated tractography in the human and macaque brain. NeuroImage. 2020 Aug 15;217:116923. doi:10.1016/j.neuroimage.2020.116923
